# Finding the missing honey bee genes: lessons learned from a genome upgrade

**DOI:** 10.1186/1471-2164-15-86

**Published:** 2014-01-30

**Authors:** Christine G Elsik, Kim C Worley, Anna K Bennett, Martin Beye, Francisco Camara, Christopher P Childers, Dirk C de Graaf, Griet Debyser, Jixin Deng, Bart Devreese, Eran Elhaik, Jay D Evans, Leonard J Foster, Dan Graur, Roderic Guigo, Katharina Jasmin Hoff, Michael E Holder, Matthew E Hudson, Greg J Hunt, Huaiyang Jiang, Vandita Joshi, Radhika S Khetani, Peter Kosarev, Christie L Kovar, Jian Ma, Ryszard Maleszka, Robin F A Moritz, Monica C Munoz-Torres, Terence D Murphy, Donna M Muzny, Irene F Newsham, Justin T Reese, Hugh M Robertson, Gene E Robinson, Olav Rueppell, Victor Solovyev, Mario Stanke, Eckart Stolle, Jennifer M Tsuruda, Matthias Van Vaerenbergh, Robert M Waterhouse, Daniel B Weaver, Charles W Whitfield, Yuanqing Wu, Evgeny M Zdobnov, Lan Zhang, Dianhui Zhu, Richard A Gibbs

**Affiliations:** 1 Division of Animal Sciences, Division of Plant Sciences, and MU Informatics Institute, University of Missouri, Columbia, MO 65211, USA; 2 Department of Biology, Georgetown University, Washington, DC 20057, USA; 3 Human Genome Sequencing Center, Department of Molecular and Human Genetics, Baylor College of Medicine, MS BCM226, One Baylor Plaza, Houston, TX 77030, USA; 4 Institute of Evolutionary Genetics, Heinrich Heine University Duesseldorf, Universitaetsstrasse 1, 40225 Duesseldorf, Germany; 5 Center for Genomic Regulation, Universitat Pompeu Fabra, C/Dr. Aiguader 88, E-08003 Barcelona, Catalonia, Spain; 6 Division of Animal Sciences, University of Missouri, Columbia, MO 65211, USA; 7 Laboratory of Zoophysiology, Ghent University, Krijgslaan 281 S2, B-9000 Ghent, Belgium; 8 Laboratory of Protein Biochemistry and Biomolecular Engineering, Ghent University, K.L. Ledeganckstraat 35, B-9000 Ghent, Belgium; 9 Department of Mental Health, Johns Hopkins University Bloomberg School of Public Health, Baltimore, MD 21205-2103, USA; 10 Bee Research Laboratory, BARC-E, USDA-Agricultural Research Service, Beltsville, MD 20705, USA; 11 Department of Biochemistry & Molecular Biology, Centre for High-Throughput Biology, University of British Columbia, 2125 East Mall, Vancouver, BC, Canada; 12 Department of Biology and Biochemistry, University of Houston, Houston, TX 77204-5001, USA; 13 Ernst Moritz Arndt University Greifswald, Institute for Mathematics and Computer Science, Walther-Rathenau-Str. 47, 17487 Greifswald, Germany; 14 Department of Crop Sciences and Institute of Genomic Biology, University of Illinois at Urbana-Champaign, Urbana, IL 61801, USA; 15 Department of Entomology, Purdue University, 901 West State Street, West Lafayette, IN 47907-2089, USA; 16 Department of Obstetrics, Gynecology & Reproductive Sciences, University of Pittsburgh, MAGEE 0000, Pittsburgh, PA 15260, USA; 17 High-Performance Biological Computing (HPCBio), Roy J. Carver Biotechnology Center, University of Illinois at Urbana-Champaign, Urbana, IL 61801, USA; 18 Softberry Inc., 116 Radio Circle, Suite 400, Mount Kisco, NY 10549, USA; 19 Institute for Genomic Biology and Department of Bioengineering, University of Illinois at Urbana-Champaign, 1270 DCL, MC-278, 1304 W Springfield Ave, Urbana, IL 61801, USA; 20 Research School of Biology, The Australian National University, Canberra ACT 0200, Australia; 21 Institut für Zoologie, Molekulare Ökologie, Martin-Luther-Universität Halle-Wittenberg, Hoher Weg 4, D-06099 Halle (Saale), Germany; 22 Genomics Division, Lawrence Berkeley National Laboratory, Berkeley, CA 94720, USA; 23 National Center for Biotechnology Information, National Library of Medicine, National Institutes of Health, Building 45, 8600 Rockville Pike, Bethesda, MD 20894, USA; 24 Department of Entomology, University of Illinois at Urbana-Champaign, Urbana, IL 61801, USA; 25 Institute for Genomic Biology, Department of Entomology, Neuroscience Program, University of Illinois at Urbana-Champaign, 1206 West Gregory Drive, Urbana, IL 61801, USA; 26 Department of Biology, University of North Carolina at Greensboro, 321 McIver Street, Greensboro, NC 27412, USA; 27 Computer, Electrical and Mathematical Sciences and Engineering Division, King Abdullah University of Science and Technology (KAUST), Thuwal 23955-6900, Kingdom of Saudi Arabia; 28 Extension Field Operations, Clemson University, 120 McGinty Ct, Clemson, SC 29634, USA; 29 University of Geneva and Swiss Institute of Bioinformatics, CMU, Michel-Servet 1, Geneva CH-1211, Switzerland; 30 Genformatic, 6301 Highland Hills Drive, Austin, TX 78731, USA; 31 Department of Entomology, Neuroscience Program, Program in Ecology and Evolutionary Biology, University of Illinois at Urbana-Champaign, Urbana, IL 61801, USA

**Keywords:** *Apis mellifera*, GC content, Gene annotation, Gene prediction, Genome assembly, Genome improvement, Genome sequencing, Repetitive DNA, Transcriptome

## Abstract

**Background:**

The first generation of genome sequence assemblies and annotations have had a significant impact upon our understanding of the biology of the sequenced species, the phylogenetic relationships among species, the study of populations within and across species, and have informed the biology of humans. As only a few Metazoan genomes are approaching finished quality (human, mouse, fly and worm), there is room for improvement of most genome assemblies. The honey bee (*Apis mellifera*) genome, published in 2006, was noted for its bimodal GC content distribution that affected the quality of the assembly in some regions and for fewer genes in the initial gene set (OGSv1.0) compared to what would be expected based on other sequenced insect genomes.

**Results:**

Here, we report an improved honey bee genome assembly (Amel_4.5) with a new gene annotation set (OGSv3.2), and show that the honey bee genome contains a number of genes similar to that of other insect genomes, contrary to what was suggested in OGSv1.0. The new genome assembly is more contiguous and complete and the new gene set includes ~5000 more protein-coding genes, 50% more than previously reported. About 1/6 of the additional genes were due to improvements to the assembly, and the remaining were inferred based on new RNAseq and protein data.

**Conclusions:**

Lessons learned from this genome upgrade have important implications for future genome sequencing projects. Furthermore, the improvements significantly enhance genomic resources for the honey bee, a key model for social behavior and essential to global ecology through pollination.

## Background

Honey bee, *Apis mellifera*, provides a key model for understanding the diverse and widespread group of social insects. Thanks, in part, to resources generated during the initial honey bee genome sequencing effort [1], honey bees are now widely used for elucidating the molecular basis of behavior [2], development [3], disease transmission [4], epigenomics [5] and gene regulation [6]. Honey bees are also a vital pollinator of many of the world’s crops [7,8], and genomic tools are being used to address recent serious and unexplained declines in some honeybee populations [9,10]. In light of the current interest in honey bee genetics, improved genomic tools for this species are required.

Because fewer than 11,000 genes were predicted, one of the major questions coming out of the initial honey bee genome sequencing effort was, “Where are the missing genes?” Were there issues with the genome sequence that meant that the genes were filled with gaps and therefore not annotated? Or were the genes located in islands of repetitive sequence and therefore not identified? Were the gene prediction methods poorly adapted to a genome with a bimodal distribution in GC content? Or was the tuning of gene prediction algorithms to an average GC content failing to represent genes in regions of more extreme GC content? Was there insufficient mRNA evidence? Were the genes different enough from known genes that the prediction tools failed to find the genes due to lack of protein evidence? Or might the honey bee have thousands fewer genes than the few insects with sequenced genomes at the time?

All early genome sequencing projects used Sanger data and either a BAC-based hierarchical-sequencing model [11] or a whole-genome-shotgun model [12]. In either case, a completed draft genome has contiguous sequence pieces (contigs) spaced by paired clone end sequences, forming scaffolds. Missing sequences between consecutive contigs are represented by segments of ambiguous bases, denoted as Ns, the lengths of estimated gap sizes. Few Metazoan genomes have been improved to finished quality by filling in the missing sequence [13-16]. The genome scaffolds, with islands of sequence information and lack of information (in a draft genome), are annotated with gene features using gene prediction methods. When gene signals such as splice sites, start codons and termination codons are missing from the assembly, a computational gene prediction tool may miss an exon or even an entire gene. Thus, gaps within genic regions of an assembly can hinder annotation and lead to an incomplete gene list.

The first *A. mellifera* genome sequencing project [1] revealed genome characteristics with potential missing assembly information that could impact the gene list. The genome assembly had the lowest mean GC content (percent of G + C nucleotides) and the most heterogeneous GC content of any sequenced metazoan genome at that time, with GC content ranging from 10% to 70% across the published genome assembly (Amel_4.0) [1]. Analyses of initial assemblies showed that regions with low GC content were under-represented in libraries, so additional shotgun libraries were generated after fractionating DNA with CsCl-bisbenzimide density gradient centrifugation, and approximately 800,000 reads of <30% GC content were added to the data that lead to Amel_4.0 [1].

In addition to potentially missing assembly information, other factors including limited EST data and the large evolutionary distance between honey bee and other insects with sequenced genomes were thought to have contributed to difficulty in gene prediction. The 78,001 *A. mellifera* ESTs that were available at the time provided only 9,408 unique consensus sequence alignments to the genome [17]. The closest organisms with available protein sets were the Dipterans, *Drosophila melanogaster* and *Anopheles gambiae*, with an estimated divergence time from honey bee (Hymenopteran) of 300 million years.

Honey bee researchers suspected that the first Official Gene Set (OGSv1.0), generated by creating a consensus gene set with GLEAN [1,17], was incomplete, as it comprised only 10,157 genes, far fewer than expected based on the estimate of ~13,600 genes in *D. melanogaster* at the time. Recently sequenced Hymenoptera genomes (parasitoid wasp and seven ant species), predicted to contain ~17,000-18,500 protein-coding genes [18-24], further support the suspicion that a large number of *A. mellifera* genes had not yet been detected. Whole genome tiling arrays provided experimental evidence for missing genes, with the detection of signals “intergenic” to OGSv1.0 genes [1]. Finally, evaluation of the genome-wide distribution of OGSv1.0 genes revealed another potential factor in computational gene prediction, which relied on algorithms optimized for gene and genome characteristics known at that time. Unlike any previously sequenced metazoan genome, OGSv1.0 genes occurred in gene regions that were more GC-poor (29% GC) than the mean genome GC content (33%), and were located in regions with GC content as low as 11% [1].

Genome sequencing and assembly methods have changed with the advent of next-generation sequencing technologies. Newer technologies typically produce shorter reads at greatly reduced cost and allow the completion of projects with much deeper sequence coverage using many more reads than a Sanger-based project. While contiguous sequence may suffer from these shorter reads that cannot span repetitive sequences, scaffolding can benefit from the increased mate-pair information. Furthermore, different sequencing technologies have different biases related to nucleotide composition, so combining technologies provides the potential for one technology to fill in gaps produced by another.

A bigger impact of the reduced sequencing cost of second-generation sequencing methods is the ability to generate much more transcript sequence than ever before. These transcript data are very useful as evidence supporting gene model prediction. As will be discussed below, the alignment of new transcriptome data provided evidence for most of the previously un-annotated genes in the honey bee genome.

This paper presents a *de novo* annotation of the *A. mellifera* genome based on an upgraded genome assembly and new transcript data. We generated additional genome sequence data using ABI SOLiD and Roche 454 paired-end sequencing technologies to superscaffold and fill gaps in the Amel_4.0 assembly using the Atlas-link software, creating the improved assembly (Amel_4.5). We also deep-sequenced the transcriptome of seven *A. mellifera* tissues and sequenced the genomes of two closely related species, the dwarf honey bee (*A. florea*) and buff-tailed bumble bee (*Bombus terrestris*) (both of which will be published elsewhere).

To avoid perpetuating errors in OGSv1.0 and avert difficulties in tracking genes between assemblies (e.g. reported in [25]), we chose not to track and update the previous annotation on the Amel_4.5 assembly. Instead we decided to annotate the Amel_4.5 assembly *de novo* and generate a new official gene set (OGSv3.2) and a new list of repetitive elements based on the most current evidence and methodologies. To learn which factors were most important in identifying previously unknown genes, and to inform potential strategies for future genome projects, we compared both gene sets and characterized genes that were missing from OGSv1.0.

## Results

### Improvements to the genome assembly

Additional sequence coverage generated using the ABI SOLiD [26] technology (~20x) and Roche 454 [27] technology (~4x) from small insert fragments (~2 k) was incorporated into Amel_4.5. The sequence data are summarized in Table 1 and Table S1 in Additional file 1 and are available from the NCBI Sequence Read Archive (SRA). We compared assembly statistics for Amel_4.5 with the previous assembly, Amel_4.0, which is described in [1] and is the assembly used by the research community since 2006. The genome assembly improvements increased the contig N50 from 40 kb to 46 kb and the scaffold N50 from 359 kb to 997 kb, with an additional 5.5% of the sequence anchored to linkage groups (Table 2).

**Table 1:** Additional sequence data for the improved honey bee genome

**Mate-pair distance**	**Roche 454 fragment**	**Roche 454 2.75 kb**	**SOLiD**
Number of reads	2.9 M	5.24 M	90 M
Read length	290 bp	56 bp	50 bp
Pairs	No	Yes	Yes
Sequence coverage	3.6×	1.3×	20×

**Table 2:** Assembly statistics for the improved honey bee genome

**Assembly**		**Number**	**N50 (kb)**^**a**^	**Bases + Gaps (kb)**	**Bases (kb)**
Amel_4.5	Anchored	340	1,209	203,000	200,000
	Scaffolds	5,644	997	250,271	229,734
	Contigs	16,501	46	229,734	229,734
Amel_4.0	Anchored	626	621/135	217,195	183,323
	Scaffolds	10,742	359	315,719	231,029
	Contigs	18,944	40	231,029	231,029

^a^For Amel_4.0 Anchored scaffolds, N50 was calculated separately for 320 oriented and 306 non-oriented scaffolds.

The completeness (extent of coverage of the genome) of the new Amel_4.5 and the earlier, Amel_4.0, assemblies were measured by BLASTN comparison to available *de novo* assembled 454 contigs from seven libraries. Amel_4.5 showed slightly more complete coverage than Amel_4.0 (88.7% vs. 88.2%; Table 3).

**Table 3:** Assembly comparison to 454 transcriptome data

**Tissue**	**Total assembled transcripts**	**Amel_4.5**		**Amel_4.0**	
		**Number**	**Percent**	**Number**	**Percent**
Abdomen	14,614	13,980	95.6%	13,987	95.7%
Brain + ovary	27,412	26,341	96.0%	26,342	96.0%
Embryo	19,616	18,565	94.6%	18,565	94.6%
Larvae	18,050	9,061	50.1%	9,041	50.0%
Mixed antennae	14,891	13,868	93.1%	13,865	93.1%
Ovary	28,451	27,500	96.6%	26,929	94.6%
Testes	10,557	9,234	87.4%	9,060	85.8%
Total	133,591	118,549	88.7%	117,789	88.2%

Note: BLAT alignments of the assembled transcripts to the genome assemblies using default parameters and counting matches of any length at 95% identity.

**Figure 1:**
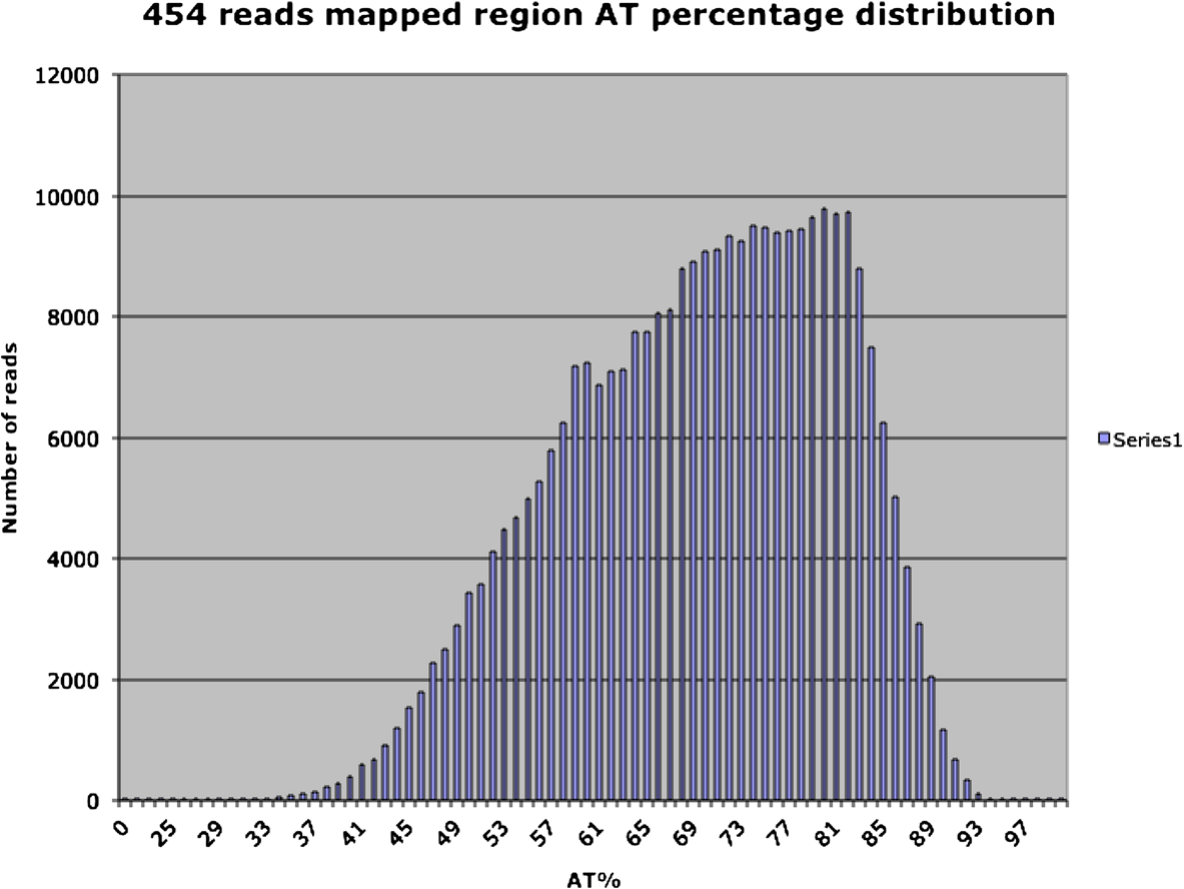
**Distribution of mapped 454 reads with respect to AT content.** The genomic reads were mapped to the Amel_4.5 assembly (scaffolds and contigs) using BLAT. With relatively stringent filtering (at least 80% of total length matched and gap size < 30%), 242,284 reads (93% of all reads) were aligned to the assembly. Most reads (236,090, 93%) aligned to fewer than 10 locations, and had unique alignments (210,625, 87%). The AT content for each alignment (adding 10% extension on either end) was calculated for reads with ≤ 10 match locations.

**Figure 2:**
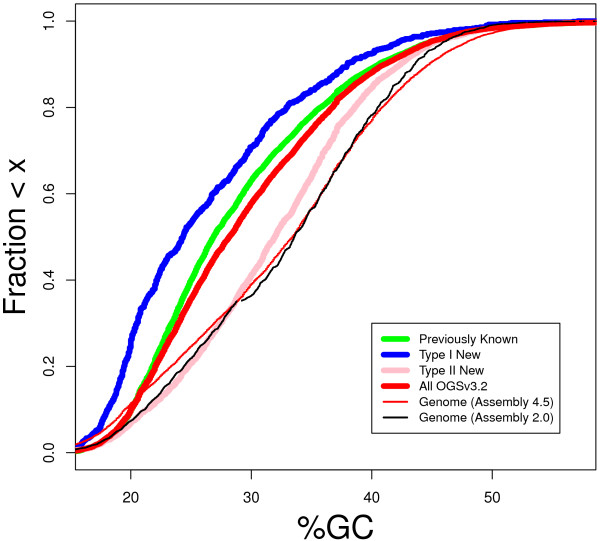
**GC content of genic regions and overall genome assemblies.** For each gene, the GC content (percent G + C nucleotides) of genomic regions containing the gene was determined as described in methods. The cumulative distributions of GC content for overall genome assemblies (thin red line for Amel 4.5 and thin black line for Amel_2.0) show that the Amel_4.5 assembly has a higher fraction of low GC content regions than does the Amel_2.0 assembly (note the thin red line is to the left of the thin black line below about 28% GC). The cumulative distributions of GC content for the regions containing genes (thick red line for all OGSv3.2, thick green line for Previously known genes, thick blue line for Type I New genes and thick pink line for Type II new genes) show that regions containing genes are lower in GC content than the overall genome. This trend applies for the complete set of OGSv3.2 genes, as well as the three subsets. The distribution for Type I New genes lies to the left of the other distributions, showing that Type I New genes are located in lower GC content regions than the other gene subsets. The distribution for Type II new genes is to the right of the distributions for the other gene subsets, showing that the Type II New genes are located in higher GC content regions.

As hoped, the new sequence data had a large impact on the regions of the genome that were of low starting quality. While the new sequence did not specifically target the GC-poor regions of the genome that were underrepresented in the initial Sanger libraries, the sequence reads were more evenly distributed across the regions of different GC contents (Figure 1). A segmentation analysis of Amel_4.5 to identify GC compositional domains that are homogenous in GC content within domains, but different in GC content across domain boundaries, showed that the genomic proportion of compositional domains low in GC content increased in Amel_4.5 (Figure 2). As a consequence, many newly discovered genes were annotated in GC poor regions (Figure 2).

### Generating and assessing a New official gene Set

#### ***Testing combined gene set approaches***

We tested two approaches, MAKER2 [28] and GLEAN [17], for combining outputs from multiple gene prediction tools to create an Official Gene Set (OGS). Each approach has advantages and disadvantages. An advantage of MAKER2 is that given sufficient transcript data, it can predict multiple alternative splice forms per locus, while GLEAN computes only one gene model per locus. MAKER2 provides both a conservative biological sequence (transcript and homolog) alignment-based set of gene models that best agree with transcript and protein homolog alignments, and a set of *ab initio* gene predictions that do not overlap the biological sequence alignment-based set.

The final set of MAKER2 biological sequence alignment-based gene set contained 12,268 genes, encoding 12,575 transcripts, with an additional set of 31,047 *ab initio* gene predictions that did not have support from transcript or protein alignments. Without filtering the *ab initio* set for likely false positives, the choices of final gene sets generated with MAKER2 would have been 1) a conservative set of only 12,268 genes, still much lower than gene numbers found in other sequenced insect genomes or 2) a set of 43,315 genes that included *ab initio* gene models. Other hymenoptera genome projects using MAKER have selected *ab initio* gene models with matches to InterPro domains [29] to include in the final predicted gene set [20-22]. This approach would allow us to add 1,215 MAKER2 *ab initio* genes to the final predicted gene set, for a total of 13,303 genes. However, since only 8,602 of the 12,268 MAKER2 biological sequence alignment-based genes had InterPro matches, we were concerned that the InterPro approach to boost *ab initio* genes to the final gene set would miss some rapidly diverging genes.

An important difference between GLEAN and MAKER2 is the way *ab initio* predictions without biological sequence evidence are handled. GLEAN collects evidence for genes (*ab initio* gene predictions and biological sequence alignment-based), generates maximum likelihood estimates of accuracy and error rates for these signals for each gene prediction set, constructs consensus gene models that maximize the scores for the signal sites in each gene model, and labels each model with a probabilistic confidence score reflecting the underlying support for that gene model [17]. In addition to identifying consensus models supported by transcript and protein alignments, GLEAN identifies *ab initio* predictions with high computed probabilities, filtering out likely false positive *ab initio* predictions. This approach would not require filtering *ab initio* predictions using an InterPro search, and would potentially include high confidence *ab initio* predictions that are true, rapidly diverging genes without significant InterPro domain matches.

Since GLEAN weighs different sources of gene prediction evidence based on agreement among sources, without *a priori* information on the quality of individual input gene prediction sets, the choice of input datasets impacts the results. Evaluating GLEAN runs with different combinations of input sets allowed us to determine the optimum selection of datasets. We ran GLEAN 32 times with different combinations of input sets, and resulting GLEAN sets ranged in size from in 10,403 (with three input gene sets) to 17,724 genes (with seven input gene sets), (Additional file 2). In some cases, GLEAN predicted a larger number of genes than the MAKER2 set supported by either biological sequence alignment or InterPro. Although GLEAN generates only one transcript per gene locus, we decided to use GLEAN to create the OGS, because we prioritized GLEAN’s potential identification of a more comprehensive set of gene loci over MAKER2’s identification of 307 alternative transcripts. Since with GLEAN we did not need to filter *ab initio* genes based on InterPro, it might allow the genome-conservation-based N-SCAN dataset to provide support for other *ab initio* predictions even without detectable conservation with known InterPro domains. Another consideration was that assembly methods for Illumina RNASeq were not well established. We were concerned about the possibility of merging genes or missing introns due to potential genomic contamination in RNASeq experiments. GLEAN uses transcript alignments to support splice predictions, but does not merge or split genes based on transcript alignments, while MAKER2 creates gene models that best agree with transcript alignments, and works best with highly reliable transcript assemblies.

#### ***Selecting an official gene set***

We evaluated the 32 GLEAN sets based on several criteria, including overlap with a conservative evidence-based set (RefSeq), transcript sequences, peptides and the CEGMA [30] conserved core set (Additional file 2). No single gene set was optimal with respect to all criteria. We chose to rank sets based on number of peptide matches, which would prioritize completeness of a protein-coding gene set rather than correctness of gene structure.

#### ***Assessing the new official gene set***

The selected GLEAN set, OGSv3.2 (GLEAN31 in Additional file 2), represented a significant improvement because it included 15,314 protein-coding genes, which is 5,157 more genes than the first official gene set, OGSv1.0. The proportion of genes on placed scaffolds as well as those with expressed sequence coverage also increased over OGSv1.0 (Table 4). Since GLEAN predicts only coding exons, but not untranslated regions (UTRs), we used MAKER2 [28] to add UTR to the final GLEAN gene predictions. Out of a total of 15,314 OGSv3.2 genes, UTR were added to 7,514 genes (49%).

**Table 4:** Comparison of OGSv1.0 and OGSv3.2

	**OGSv1.0**	**OGSv3.2**
Number of genes	10,157	15,314
Number of genes within mapped scaffolds (% of total no. of genes)	5,973 (58.8%)	13,285 (86.8%)
Number of genes within un-mapped scaffolds (% of total no. of genes)	4,184 (41.2%)	2,029 (13.2%)
Average coding sequence length (bp)	1,623	1,266
Average number of coding exons	6.4	5.3
Number of single coding exon genes (% of total no. of genes)	795 (7.8%)	2,059 (13.4%)
Number of multi-coding exon genes (% of total no. of genes)	9,362 (92.2%)	13,255 (86.6%)
Number of genes with spliced EST coverage (% of total no. of genes)	3,039 (29.9%)	12,172 (79.5%)
Number of genes with un-spliced EST coverage (% of total no. of genes)	1,734 (17.1%)	11,019 (72%)
Number of genes that overlap a protein alignment (% of total no. of genes)	7,940 (78.2%)	6,778 (44.3%)

Many split and merged gene models were noted when comparing the 32 GLEAN sets, including OGSv3.2, to the conservative RefSeq gene set (Additional file 2). Since it is difficult to computationally resolve disagreements among gene sets, the OGSv3.2 genes in question are provided as a separate track on the BeeBase Amel_4.5 genome browser [31]. Web Apollo [32] annotation tools allow registered BeeBase users to modify gene models, and can be used to manually correct split or merged genes [33].

**Figure 3:**
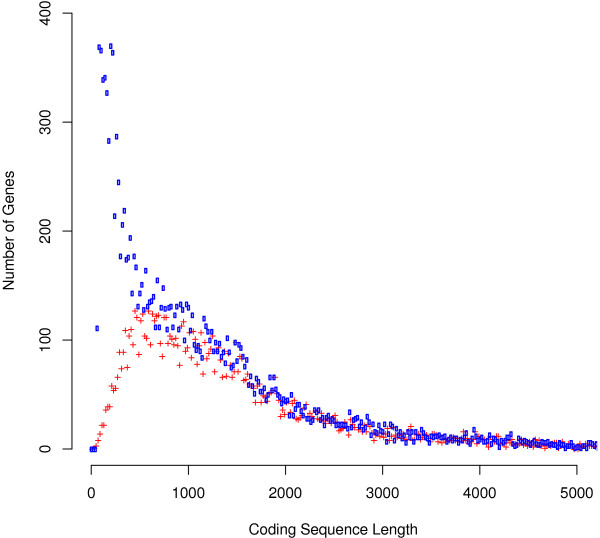
**Distribution of coding sequence lengths in OGSv3.2 and OGSv1.0.** Histogram plots showing the number of genes having “X” coding sequence length in bins of 20 nt are illustrated using points instead of lines to allow visualization of both distributions. The range in coding sequence length extends to 70,263 and 53,649 in OGSv3.2 (blue) and OGSv1.0 (red), respectively, but this figure zooms in to show lengths only up to 5,000 nt. There were 386 and 344 genes with coding sequences longer than 5,000 nt in OGSv3.2 and OGSv1.0, respectively. This figure shows that the increased number of genes in OGSv3.2 is largely due to increased numbers of short genes. The number of larger genes is not decreased, so gene splitting is not likely a major source of additional genes.

To address the question of whether the increased gene number was due to splitting genes, we looked at the total number of coding nucleotides (nt) and the distribution of coding sequence lengths in OGSv1.0 and OGSv3.2. The ranges in coding sequence lengths were 24-53,649 and 75-70,263 nt for OGSv1.0 and OGSv3.2, respectively. Note that the gene prediction parameters did not allow coding sequences less than 75 nt for OGSv3.2, but a minimum coding sequence parameter was not applied in the generation of OGSv1.0; however there were only 6 OGSv1.0 genes less than 75 nt. The OGSv3.2 gene with the largest coding sequence overlapped a single RefSeq gene prediction (XM_623650.3), and was found to be homologous to genes predicted to code for titin. The total number of coding nucleotides increased from 16,484,776 in OGSv1.0 to 19,342,383 in OGSv3.2. The increase in coding nucleotides by only 2,857,607 (17.3%) compared to a 50.8% increase in the number of genes suggested that new genes were shorter and possibly a result of splitting OGSv1.0 gene models. However, the distribution of coding sequence length in the two gene sets suggested that splitting genes was not the major source of the increased gene number (Figure 3). Although OGSv3.2 possessed a large number of short coding sequences (< ~1.5 kb) compared to OGSv1.0, the larger genes did not appear to be missing. Furthermore, analyzing overlapping gene alignments (described below), showed that 4,735 of the OGSv3.2 genes did not overlap with genes in OGSv1.0, and thus were not likely to be a result of splitting OGSv1.0 genes.

#### ***Biological evidence for the new official gene set***

In assessing the gene set, we define biological evidence to include biological sequence alignments used in gene prediction pipelines (transcript, peptide, protein homolog) as well as matches to InterPro domains and alignment to other bee genomes. We define biological *gene* evidence as all of the datasets considered as biological evidence, except alignment to other bee genomes, since we did not use information about genes or expression for the other bee genomes. Some form of biological gene evidence supported the majority (14,084, 92%) of the OGSv3.2 genes. An additional 754 (4.9%) OGSv3.2 genes aligned to either the *Apis florea* or *Bombus terrestris* genome for a total of 14,836 (96.9%) of the OGSv3.2 genes with biological support. Requiring that transcripts be spliced reduces the number of supported genes to 13,264 (86.8%) or 14,661 (95.7%) if alignment of the gene to another bee genome is considered as support.

### Identification and characterization of New genes

#### ***Identifying new genes due to improved assembly or improved gene prediction***

In order to compare the old and new OGS (OGSv1.0 and OGSv3.2), we mapped OGSv3.2 coding sequences to the Amel_2.0 assembly because it was the assembly used to predict OGSv1.0 genes [1], which were later mapped to Amel_4.0. We chose to use Amel_2.0 in this step, because generation of Amel_4.0 and intermediate assemblies had involved rearranging and splitting scaffolds and incorporating unscaffolded contigs using an updated genetic map [1]. We wished to minimize mapping errors in our comparison of gene sets by using Amel_2.0 to determine which OGSv3.2 genes correspond to OGSv1.0 genes.

We performed the mapping twice using different alignment criteria, stringent (95% identity and 80% alignment coverage) and relaxed (95% identity and 50% alignment coverage). We will report results only for the stringent criteria (Table 5) unless otherwise specified, but have provided the relaxed mapping results in Table S2 in Additional file 1. On the basis of mapping to Amel_2.0 and overlap between the OGSv3.2 coding sequence alignments with OGSv1.0 gene models, we divided the 15,314 OGSv3.2 genes into three sub-sets (Table 5). Any OGSv3.2 gene that did not align to the Amel_2.0 assembly we deemed a “Type I New” gene. Any OGSv3.2 gene that aligned to the Amel_2.0 assembly, but whose coordinates did not overlap an OGSv1.0 gene by a single coding base pair on the same strand, we deemed a “Type II New” gene. Finally, any OGSv3.2 gene that both aligned to the Amel_2.0 assembly and overlapped an OGSv1.0 gene we deemed a “Previously Known” gene.

**Table 5:** New and previously known OGSv3.2 genes

		**All OGSv3.2**	**Type I new genes**	**Type II new genes**	**Previously known genes**
	Number of genes (% of total OGSv3.2 genes)	15,314 (100%)	782 (5.1%)	3,953 (25.8%)	10,579 (69.1%)
Scaffold analysis	Number of genes within mapped scaffolds (% of no. of gene type)	13,285 (86.8%)	544 (69.6%)	3,199 (80.9%)	9,542 (90.2%)
	Number of genes within un-mapped scaffolds (% of no. of gene type)	2,029 (13.2%)	238 (30.4%)	754 (19.1%)	1,037 (9.8%)
CDS analysis	Average CDS length	1,266	1,172	330	1,622
	Average no. CDS Exons	5.3	5.6	2.1	6.5
	Number of single CDS exon genes (% of no. of gene type)	2,059 (13.4%)	101 (12.9%)	1,239 (31.3%)	719 (6.8%)
	Number of multi-CDS exon genes (% of no. of gene type)	13,255 (86.6%)	681 (87.1%)	2,714 (68.7%)	9,860 (93.2%)
Intron analysis	Number of introns (% of total OGSv3.2 introns)	66,212 (100%)	3,585 (5.4%)	4,333 (6.5%)	58,294 (88%)
	Number of introns validated by EST intron coordinates (% of introns of gene type)	54,514 (82.3%)	2,573 (71.8%)	1,930 (44.5%)	50,011 (85.8%)
Peptide analysis	Number of genes with a peptide match (% of no. of gene type)	3,631 (23.7%)	132 (16.9%)	82 (2.1%)	3,417 (32.3%)
Protein analysis	No. of genes with overlap to at least one protein alignment (% of no. of gene type)	6,778 (44.3%)	270 (34.5%)	186 (4.7%)	6,322 (59.8%)
	No. of genes with overlap to a Dmel protein alignment (% of no. of gene type)	1,205 (7.9%)	38 (4.9%)	13 (0.3%)	1,154 (10.9%)
Total spliced and un-spliced expressed sequence support	No. of genes with overlap to at least one transcript alignment from any of the ten libraries (% of no. of gene type)	13,517 (88.3%)	704 (90.0%)	2,771 (70.1%)	10,042 (94.9%)
Spliced expressed sequence analysis	No. of genes with overlap to at least one spliced transcript alignment from each of the ten libraries (% of no. of gene type)	1,062 (6.9%)	32 (4.1%)	15 (0.4%)	1,015 (9.6%)
	No. of genes with overlap to at least one spliced transcript alignment from any of the ten libraries (% of no. of gene type)	12,172 (79.5%)	622 (79.5%)	2,110 (53.4%)	9,440 (89.2%)
	No. of genes without overlap to any spliced transcript alignments in any of the ten libraries (% of no. of gene type)	3,142 (20.5%)	160 (20.5%)	1,843 (46.6%)	1,139 (10.8%)
	Genes broadly expressed across four tissues (% of no. of gene type)	2,326 (15.2%)	60 (7.7%)	95 (2.4%)	2,171 (20.5%)
	Genes narrowly expressed in only a single tissue (% of no. of gene type)	3,346 (21.8%)	234 (29.9%)	1,139 (28.8%)	1,973 (18.7%)
	No. of genes without overlap to any spliced transcript alignments in any of the four tissues (% of no. of gene type)	3,632 (23.7%)	192 (24.6%)	1,985 (50.2%)	1,455 (13.8%)
Analysis of alignments to other bee genomes	No. of genes that align to Aflo_1.0 (% of no. of gene type)	13,491 (88.1%)	566 (72.4%)	2,584 (65.4%)	10,341 (97.8%)
	No. of genes that align to Bter_1.0 (% of no. of gene type)	12,262 (80.1%)	527 (67.4%)	1,566 (39.6%)	10,169 (96.1%)
Evidence-supported genes	No. of genes with overlap to at least one form of biological evidence (% of no. of gene type)	14,084 (92.0%)	713 (91.2%)	2,930 (74.1%)	10,441 (98.7%)
	No. of genes that align to Aflo_1.0 and/or Bter_1.0 and/or overlap at least one form of biological evidence (% of no. of gene type)	14,836 (96.9%)	734 (93.9%)	3,555 (89.9%)	10,547 (99.7%)
GC analysis	Number of genes on GC compositional domains >10 kb (% of OGSv3.2 total)	15,224 (99.4%)	777 (5.1%)	3,923 (25.8%)	10,524 (69.1%)
	Avg. GC content of compositional domain gene resides in	29.60%	26.40%	32.00%	28.90%
ENC analysis	Effective number of codons	44.95	41.97	45.69	44.9

Genes were mapped to Amel_2.0 assembly with stringent mapping criteria of 80% gene coverage and 95% identity. Biological evidence includes transcript overlap (spliced or un-spliced), peptide hit, protein homolog alignment overlap, or InterPro domain presence.

Of the 5,157 additional genes in OGSv3.2 compared to OGSv1.0, 4,735 genes did not overlap OGSv1.0 genes. The other 422 additional genes in OGSv3.2 were due to either splitting OGSv1.0 genes (discussed above) or to the annotation of additional recent paralogs in OGSv3.2, and are included in the total count of 10579 Previously Known genes. The 4735 genes that did not overlap with OGSv1.0 genes could be classified as 782 genes discovered due to the additional sequencing and reassembly of the bee genome for the Amel_4.5 assembly (Type I New genes; Table 5) and 3,953 genes detected by improved gene prediction, either through the use of new expressed sequence and protein data or improved gene prediction algorithms (Type II New genes; Table 5). We could map 405 additional Type I New genes to the Amel_2.0 assembly if we required only 50% of the gene to be covered (Table S2 in Additional file 1), rather than the more stringent 80% gene coverage reported in Table 5, consistent with the Amel_2.0 assembly being less continuous than Amel_4.5. This lack of contiguity and resulting fragmentation of genes in the Amel_2.0 assembly likely impaired the initial gene prediction efforts. While the frequency of genes with some form of biological support (transcript, peptide, protein homolog, InterPro domain, and/or alignment to another bee genome) was highest for Previously Known genes (99.7%), most of the Type I New genes (93.8%) and Type II New genes (89.9%) were also supported (Table 5).

#### ***Characteristics of new genes***

We analyzed the data that went into the annotation of OGSv3.2 and evaluated which pieces of evidence contributed to the prediction of the genes to understand why they were missed in OGSv1.0. To determine whether genes that were not detected in OGSv1.0 have common characteristics that make them more challenging to predict, we compared them to the Previously Known genes. We evaluated features such as tissue expression specificity, coding feature length, GC content, overlap of protein homolog alignments on Amel_4.5 and the proportion of non-canonical splice sites. Additional file 3 provides sources of evidence for each OGSv3.2 gene.

The mean coding sequence lengths of Type I New (1,172 bp) and Type II New genes (331 bp) were shorter than that of Previously Known genes (1,623 bp) (P = 2.3 × 10^-12^ and P < 2.2 × 10^-16^ respectively) (Table 5). This difference may be due to a higher fraction of single coding exon genes among new genes. Thirteen percent of Type I New genes and 31% of Type II New genes contained one coding exon, while only 6.8% of Previously Known genes contained one coding exon (P < 2.805 × 10^-10^ and P < 2.2 × 10^-16^ for Type I and Type II genes, respectively) (Table 5). The number of canonical versus non-canonical splice sites was not significantly different between the Previously Known and Type II New genes (Table S3 in Additional file 1).

Type I New genes were found to reside in GC compositional domains with mean 26.4% GC, significantly lower than that of Previously Known genes (28.9%) (P = 2.188 × 10^-13^), supporting improvement in the assembly of the high-AT-content regions. On the other hand, Type II New genes were found in GC compositional domains with a mean GC content of 32.0%, higher than that of than Previously Known genes (P < 2.2 × 10^-16^), but still slightly lower than the mean GC content of the genome (32.7%)

The effective number of codons is an estimate of the deviation from equality of synonymous codon usage of all codons of a gene, and ranges from 20 (extreme bias where only one codon is used for each amino acid) to 61 (no bias, all synonymous codons are used equally) [34]. Type I New genes had a significantly lower mean effective number of codons than Previously Known genes, 41.97 vs. 44.90 respectively (P = 2.26 × 10^-12^) (Table 5). This is consistent with the idea that the more extreme the deviation from equal proportions of G + C and A + T nucleotides in coding sequences, the lower the potential diversity of synonymous codons. Consistent with their locations in less extreme GC compositional domains, Type II New genes had a significantly higher mean ENC than the Previously Known genes, 45.69 vs. 44.90 respectively (P = 3.923 × 10^-05^) (Table 5).

#### ***Expression evidence for new genes***

The majority of OGSv3.2 genes were supported by transcript evidence. When combined, the spliced and un-spliced transcript alignments overlapped 13,517 (88.3%) of OGSv3.2 genes. An analysis of OGSv3.2 gene coverage by transcript dataset (Table S4 in Additional file 1) showed that the fraction of genes represented in a transcript dataset ranged from 28.3% (both larvae and testes) to 79.2% (forager brain) (Table S4 in Additional file 1). Both Type I New (79.5%, 622) and Type II New (53.4%, 2110) genes were less likely to overlap a spliced transcript alignment than Previously Known genes (89.2%, 9,440) (P = 3.298 × 10^-16^ and P < 2.2 × 10^-16^, respectively) (Table 5).

Compared to Previously Known genes both Type I New and Type II New genes were more likely to be narrowly expressed or overlap transcript alignments from only one tissue (P = 2.136 × 10^-14^ and P < 2.2 × 10^-16,^, respectively) 18.7% of Previously Known, 29.9% of Type I New and 28.8% of Type II New were narrowly expressed (Table 5). Conversely, Previously Known genes (20.5%, 2,171) were more likely to be broadly expressed or overlap transcript alignments from all tissues than either Type I New or Type II New genes (P < 2.2 × 10^-16^ for both tests). 20.5% of Previously Known, 7.7% of Type I New and 2.4% of Type II New genes overlapped transcript alignments from all tissues. However, the fractions of narrowly expressed (18.7%) and broadly expressed (20.5%) genes were similar to each other for Previously Known genes.

#### ***Homolog alignment evidence for new genes***

Results suggested that the use of dipteran proteins as the main source of protein homolog evidence for OGSv1.0 may have been a contributing factor to an incomplete gene set; 44.3% of the OGSv3.2 genes overlapped at least one protein homolog alignment, but only 7.9% overlapped an alignment of a *D. melanogaster* protein (Table 5). Differences among gene sets further supports the limited value of Dipteran proteins as a primary source of homolog evidence for *A. mellifera* gene prediction. Both Type I New and Type II New genes were less likely than Previously Known genes to overlap a *D. melanogaster* homolog alignment (P = 1.394 × 10^-07^ and P < 2.2 × 10^-16^, respectively). The effect was more drastic for Type II New genes; only 0.3% of Type II New genes overlapped a *D. melanogaster* homolog alignment, while the proportions were 4.9% and 10.9% for Type I New and Previously Known genes, respectively.

Both Type I New and Type II New genes were less likely than Previously Known genes to overlap any homolog alignment from another sequenced genome (P < 2.2 × 10^-16^ for both tests), but the difference was more extreme for Type II New genes, potentially implying that a greater number of the Type II New genes are specific to either *A. mellifera*, bees, or Hymenoptera than the Previously Known genes (Table 5). 59.8% of Previously Known genes overlapped a homolog alignment, while only 34.5% and 4.7% of Type I and Type II new genes, respectively, overlapped a homolog alignment (Table 5).

#### ***Genome alignment evidence for new genes***

Over 80% of the OGSv3.2 genes aligned to both the Aflo_1.0 and Bter_1.0 genome assemblies (Table 5). A notable difference between Type II New genes and the other gene sets was the proportion of genes that were supported by genome conservation, but not other sources of evidence. Of the 3,555 Type II New genes supported by any evidence, 17.5% were not supported by other sources. On the other hand, only 2.9% of supported Type I New genes and 1% of supported Previously Known genes were supported by only genome conservation.

The remaining 20% of the OGSv3.2 genes have the potential to be *Apis*-specific (8% of genes that align to Aflo_1.0, but not Bter_1.0) or *A. mellifera* specific (12% of OGSv3.2 genes that do not align to Aflo_1.0) (Table 5).

### Peptide analysis

#### ***Use of peptide evidence***

Peptide data were used in four ways. First, peptide data were used in gene prediction by AUGUSTUS, the only program used in this study that accepts this source of gene evidence. Second, peptides were compared to all consensus gene prediction sets to identify the set with the highest representation of peptides (described above; Additional file 2). Third, peptide support was used in characterizing Previously Known, Type I and Type II New genes. Fourth, venom peptides were used to manually annotate venom genes associated with the sting of the honey bee.

#### ***Gene set comparison with all peptides***

Peptide sequences aligned to a greater number of Previously Known genes than to Type I or Type II New genes (P < 2.2 × 10^-16^ for both tests). Peptides aligned to 32% of the Previously Known genes, but to only 17% of Type I and 2% of Type II New genes (Table 5 and Additional file 3).

#### ***Venom peptide analysis***

Despite the lower proportion of new genes compared to Previously Known genes with peptide matches, analysis of venom peptides (a subset of the peptide data) showed that the Amel_4.5 assembly provides a significant contribution to venom proteome research. 705 unique venom peptides provided biological evidence for 102 genes (described in [35]). Searching the venom mass spectra against OGSv1.0 and OGSv3.2 showed that the improved assembly allowed detection of 21 additional peptides supporting 9 new venom protein identifications in OGSv3.2 (Additional file 4). Additional tryptic peptides were discovered for 7 venom proteins as a result of improved gene predictions (Additional file 4).

Manual annotation of genes supported by the venom peptides (Additional file 5) using Apollo [36] showed that most honey bee venom genes are fully (76.5%) or partially (19.6%) covered by transcriptome evidence. The putative biological functions of these genes are described elsewhere [35]. We discovered that one of the annotated genes (GB40695) codes for tertiapin. While the tertiapin peptide was already found in bee venom [37], no genomic or transcriptomic evidence had been described, an issue which is now solved as the genome improvement project supplies both a gene prediction (GB40695, NCBI Gene ID 100576769) and EST evidence (Genbank:HP466647.1). The gene is positioned on linkage group 12, next to the apamin and mast cell degranulating peptide venom genes. The three genes are tandemly arranged which suggest their origin by gene duplication via unequal crossing over, and may also point to a joint control of transcription [38].

### Orthology assessment

Analysis of two different *A. mellifera* gene sets in comparison to genes from other insects allowed us to investigate the impact of the new genome and gene annotations on the numbers of *A. mellifera* genes in near-universal orthologous groups. Although true gene losses can and do occur, these near-universal insect orthologous groups highlight possible missed gene annotations in each species.

**Figure 4:**
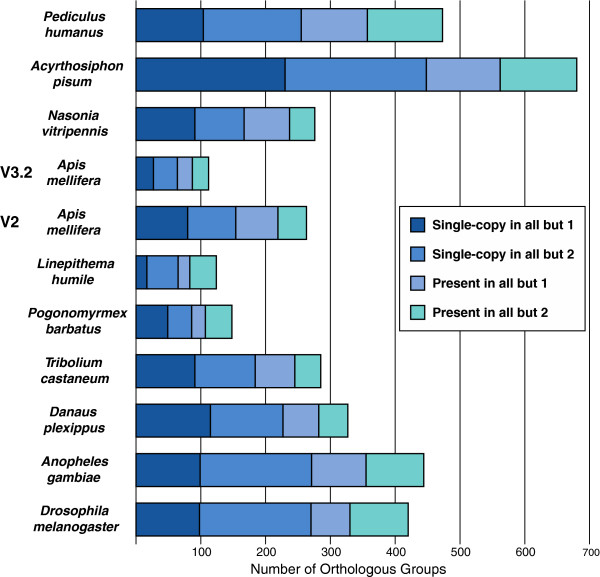
**Insect orthologs in two *A. mellifera *gene sets (V2 and OGSv3.2).** For each species, counts of near-universal orthologous groups that are missing an ortholog in that species, or in that species and one other species, are shown. Total counts are divided into groups with only single-copy orthologs and those with gene duplications, further divided into those with only one missing species and those with two missing species.

Numbers of orthologs were counted for each of the two sets, V2 (similar to OGSv1.0, described in Methods) and OGSv3.2. Figure 4 and Table S5 in Additional file 1 show the near-universal orthologs missing in each genome. The pea aphid (*Acyrthosiphon pisum*) was found to be missing more orthologous groups than the other insects. The *A. mellifera* V2 annotation was average for the genomes analyzed (missing 263 orthologs), but the OGSv3.2 annotation was much more complete, missing fewer orthologs (112) than the other genomes. Thus, the new gene set OGSv3.2 reduced the total number of potentially missing orthologs in *A. mellifera* by 57%, demonstrating the annotation improvement that recovered more “universal” insect orthologs than the previous one did.

### Predicted gene functions

#### ***Gene ontology analysis***

Gene Ontology analysis (Additional file 6) showed enrichment of some specific functions in new genes. Type I New genes were enriched for the biological processes “apoptosis” (corrected e-score = .0016), “neurotransmitter release” (corrected e-score = .0025), and “secondary active organic cation transmembrane transporter” (corrected e-score < .0001). The detection of these Type I New genes due to new assembly data is likely due to their location in low GC content regions, which were underrepresented in the older assembly; this is in agreement with the lower mean GC content of GC compositional domains containing Type I New genes. Type II new genes were enriched for the molecular function “nuclease activity” (corrected e-score = .011). Rapidly evolving and single exon genes are more difficult to annotate automatically, so it is perhaps not surprising that we found significant differences in some activities.

#### ***InterPro analysis***

Comparison of InterPro protein domains found in OGSv3.2 and OGSv1.0 proteins showed that 269 of the InterPro domains present in OGSv3.2 were not present in OGSv1.0 (Additional file 7). There were also significant differences in the fraction of genes with the domains IPR004117 (Olfactory receptor, Drosophila, p < .02) and IPR001888 (Transposase, type 1; p < .01). There were 141 and 92 genes with olfactory receptor domains in OGSv3.2 and OGSv1.0, respectively. Olfactory receptor genes contain a single olfactory receptor domain, so the domain count corresponds to the gene count. The additional olfactory receptor genes were found among both the Type I and Type II New genes. The family of olfactory receptor genes is expanded in hymenopteran insects, with rapidly diverging family members that are often arranged in tandem arrays [20,21,39]. Many of the 166 known *A. mellifera* olfactory receptor genes were identified by manual annotation of the previous assembly, because they were not found in OGSv1.0 [20,21,39]. Improved computational prediction of olfactory receptors in the new assembly was likely due to increased assembly continuity which would improve identification of tandemly repeated genes, and new sources of biological sequence alignment evidence (e.g. transcript and protein homolog sequences) which improve identification of rapidly diverging genes.

There were 18 and 3 genes with transposase type 1 domains in OGSv3.2 and OGSv1.0, respectively. Thirteen of the additional transposase type 1domains were in Type 1 New genes. Transposases are associated with transposable elements, which are a class of interspersed repeats. Since the new assembly information filled in gaps, and gaps in *de novo* assemblies are found where the assembly process fails to find an unambiguous path (e.g. repetitive loci), we were not surprised to identify InterPro domains associated with repetitive sequences among the Type I New genes. Predicted genes with matches to transposable element domains are often removed from gene sets. We further investigated OGSv3.2 for presence of interspersed repeats, and decided not to remove the small number of genes associated with transposable element domains, because evidence suggested that some were real host genes (see Genome-wide repeat analysis).

### Genome-wide repeat analysis

Processing the Amel_4.5 assembly with the REPET pipeline yielded 2,401 *de novo* predicted repetitive elements, of which 1,045 were validated by annotation of at least one complete copy. In total 9.46% (22.13 Mb) of the genome appears to be repetitive (Table 6). Non-interspersed repeats (SSR, low complexity, satellite) accounted for 4.05% (9.49 Mb), whereas interspersed repeats represented 5.07% (12.69 Mb) of the Amel_4.5 assembly. The latter estimate for transposable elements is similar to a previous estimate of 3% [1], whereby only *Mariner* and *R2* elements were reported. Thus the honey bee remains a species with an unusually low amount of repetitive DNA.

**Table 6:** **Repetitive elements in the *Apis mellifera *** **genome**

**Type of element**	**No. elements (no. chimeric/nested insert)**^**a**^	**No. Ffagments (no. full length copies)**^**b**^	**Genome coverage (bp)**	**% of genome (234.087 Mb)**	**A**^**c**^	**B**^**c**^	**C**^**c**^	**D**^**c**^	**E**^**c**^
**Repetitive DNA**			22,134,229	9.46					
***NON-INTERSPERSED REPEATS***			94,86,745	4.05					
SSR	29,697		1,441,651	0.62					
Low complexity	31,728		8,001,104	3.42					
Satellite	5 (0)	75 (6)	43,990	0.02	0	na	0	0	0
***INTERSPERSED REPEATS***	881 (65)	28,004 (1102)	12,647,484	5.40	25	7	40	2	6
***Class I - Retrotransposons***	758 (13)	21,244 (903)	9,790,204	4.18	4	1	4	0	0
**LTR retrotransposons**	2 (9)	42 (4)	49,549	0.02	1	1	1	0	0
Copia	1 (3)	29 (3)	43,892	0.02	0	1	1	0	0
Gypsy	0 (2)	0 (0)	0	0.00	0	0	0	0	0
Bel-Pao	0 (4)	0 (0)	0	0.00	0	0	0	0	0
Unclassified LTR retrotransposons	1 (0)	13 (1)	5,657	0.00	1	0	0	0	0
**DIRS retrotransposons**	2 (0)	9 (3)	12,472	0.01	0	0	2	0	0
**LINE (non-LTR) Retrotransposons**	3 (4)	140 (3)	83,103	0.04	2	0	1	0	0
R2 (NeSL, R2, R4, CRE)	2 (1)	112 (2)	72,107	0.03	2	0	0	0	0
Jockey (Rex, Jockey, Cr1, Kiri, L2, crack, Daphne)	0 (1)	0 (0)	0	0.00	0	0	0	0	0
I (R1, I, Nimb, outcast, Tad, Loa)	1 (1)	28 (1)	10,996	0.00	0	0	1	0	0
Unclassified LINE	0 (1)	0 (0)	0	0.00	0	0	0	0	0
**SINE**	19 (0)	222 (29)	69,938	0.03	0	0	0	0	0
SS-Sine	5 (0)	31 (7)	22,660	0.01	0	na	0	0	0
Unclassified SINE	14 (0)	191 (22)	47,278	0.02	0	na	0	0	0
**Unclassified retrotransposons**	1 (0)	2 (1)	8,526	0.00	0	na	0	0	0
**LARD**	301 (0)	16,406 (348)	7,256,932	3.10	1	0	0	0	0
**TRIM**	430 (0)	4,423 (515)	2,309,684	0.99	0	0	0	0	0
***Class II - DNA transposons***	51 (52)	3,209 (93)	1,339,131	0.57	7	6	27	2	5
**TIR**	50 (46)	3,200 (89)	1,335,380	0.57	7	6	27	2	5
Tc1/Mariner	43 (40)	2,636 (80)	1,147,521	0.49	5	6	25	2	5
PiggyBac	2 (6)	184 (2)	87,963	0.04	2	0	2	0	0
Unclassified TIR DNA transposons	5 (0)	380 (7)	99,896	0.04	0	na	0	0	0
**Unclassified DNA-transposons**	0 (6)	0 (0)	0	0.00	0	0	0	0	0
**MITE**	1 (0)	9 (4)	3,751	0.00	0	na	0	0	0
***Unclassified, putative elements***	72 (0)	3,551 (106)	1,518,149	0.65	14	na	9	0	1
**Other DNA elements (not repetitive DNA)**	158 (0)	13,760 (250)	6,934,063	2.96	17	0	0	0	0
Not categorized	6 (0)	946 (11)	1,233,884	0.53	0	na	0	0	0
Potential host gene^d^	152 (0)	12,814 (239)	5,700,179	2.44	17	na	0	0	0

For each group, the number of elements (putative families), the number of element fragments or copies in the genome, the cumulative length, the proportion of the genome and other features (elements containing chimeric or nested inserts of other elements (A), elements that appear to be complete with all typical structural and coding parts present even if stop codons or frameshifts are present (B), elements with a RT or *Tase *domain detected (C), potentially active elements that contain an intact ORF with all the typical domains although these can lack terminal repeats (D), elements with an intact ORF for the RT domain or parts of the *Tase *domain that could thus be partly active (E) are shown. The elements that could not be categorized or contained features of *A. mellifera* coding regions are shown at the bottom, these are probably not transposable elements.

^a^The numbers of chimeric/nested elements within elements of other categories are not included in the total numbers of elements.

^b^The software uses alignments to identify the longest fragment, which it deems as full-length. The number of full-length copies is also included in the total number of fragments.

^c^Additional Columns:

A. No. elements containing inserts

B. No. complete elements

C. No. elements with RT or Tase domains

D. No. potentially active elements

E. No. potentially partially active elements

^d^Potential host genes were predicted by software using DNA characteristics, not by overlap analysis with gene predictions. An example of a potential host gene element is a coding sequence for a repeated protein domain or motif.

Most of the groups of retrotransposable elements were detected in the genome of the honey bee. In comparison to many other organisms, the most striking difference is the extremely low diversity and abundance of these elements. LTR retrotransposons accounted for only 0.02% of the genome (49.6 kb) and included examples from only one *Copia* and a putative, unclassified element. Only fragments of elements from the *BelPao* and *Gypsy* superfamily were found. The DIRS group was represented by two incomplete elements and accounted for 0.01% of the genome (12.5 kb). LINEs accounted for only 0.04% of the genome (83.1 kb), and were represented by only two *R2 elements,* one *I* (*nimbus*) element, and a few fragments, potentially belonging to *I* (*R1*) and Jockey (*CR1*). Of the SINE elements detected, 14 could not be classified and five had similarities to SINEs of the 5S type, all representing 0.03% of the genome (70 kb). Together with another unclassified element, all class I retroelements summed up to only 224 kb (0.09%) of the genome, among the lowest in the animal kingdom. TRIMs (terminal repeat retrotransposons in miniature) [40] and LARDs (large retrotransposon derivatives) [41] are derivatives of retroelements and were detected in larger number occupying 9.57 Mb (4.09%) of the genome (Table 6).

Class II DNA transposons were more frequent and accounted for 0.57% of the genome (1.34 Mb). The vast majority of the elements were TIR (terminal inverted repeat) transposons of the *Mariner* superfamily (0.49%, 1.15 Mb). Otherwise only two elements of the *PiggyBac* superfamily (0.04%, 88 kb) and five unclassified TIR elements could be detected. Other types of DNA transposons, *Crypton*, *Maverick* and *Helitron* could not be found. The DNA transposon derivatives, MITES (miniature inverted transposable elements), were found only once and accounted for less than 0.01% of the genome (3.8 kb) (Table 6).

Besides the well-classified sequences, many repetitive elements could not be assigned to a superfamily or even class. The latter includes a larger number of elements (0.65%, 1.52 Mb), which could represent novel types, but need further investigation. We separately annotated and excluded from the transposable element counts elements that could not be categorized and elements without typical transposable element features that contained profiles from protein coding genes. Both together comprise 2.96% (6.93 Mb) of the genome (Table 6).

The detected elements of the *R2* and the majority of those of the *Mariner* type belonged to or were very similar to previously described elements from *A. mellifera* or other insects (RepBase v17.01, [42]). A few other *Mariner* and the *PiggyBac* elements showed similarities to elements known from distant animal species. This indicates a potential horizontal gene transfer as previously suggested [43].

Most of the elements appeared to be fragmented and incomplete. Although some contained sequences of typical transposable element protein domains, they seemed to be inactive due to stop codons and frameshift mutations. We detected only four retrotransposons and 27 DNA transposons with RT (reverse transcriptase) or *Tase* (transposase) domains, respectively. None of the retrotransposons appeared to possess an active ORF containing an entire RT domain, so we classified them as inactive. Among the DNA transposons, six of the *Mariners* appeared to be complete and two were potentially active. Five additional *Mariner* elements possessed an intact ORF spanning at least parts of a *Tase* domain, so they might have limited activity. The higher abundance and higher number of chimeric inserts of *Mariners* (Table 6) suggests that transposons were more recently active than retrotransposons.

### Repeats associated with the official gene set

Since gene annotation had been performed on an assembly that was masked for repeats early in the project, prior to the availability of results from the genome-wide repeat analysis, we expected some OGS genes to overlap newly-detected repeats due to incomplete masking. Coding sequences of 1,234 genes overlapped interspersed repeats detected by REPET. These genes were further investigated for characteristics that would support their annotation as host genes, including whether the gene was Previously Known, had multiple coding exons, overlapped a spliced transcript alignment, and had InterPro domain matches (Additional file 8). Of these 1,234 REPET-overlapping genes, 739 were classified as Previously Known, 185 as Type I New genes, and 310 as Type II New genes. 1,040 of the REPET-overlapping genes had multiple exons and 972 overlapped spliced transcript alignments.

Inspecting InterPro results to identify protein domains known to be associated with transposable elements, but not host genes, resulted in the following list of domains: DDE superfamily endonuclease; Integrase, catalytic core; Reverse transcriptase, RNA-dependent DNA polymerase; Retrotransposon, Pao; Ribonuclease H domain; Ribonuclease H-like domain; Transposase, Tc1-like; Transposase, type 1; Transposase, Synechocystis PCC 6803. Of the 760 REPET-overlapping genes that had InterPro matches, only 35 matched one of these transposable element domains. Some protein domains, such as zinc fingers, peptidases and helicases, are similar between host genes and transposable elements, so cannot be used to classify genes as transposable elements. Among the genes that matched non-transposable element domains were some known to be members of large gene families, such as olfactory receptors, and genes with repetitive domains, such as ankyrin repeat-containing domains. We were not surprised that these would be included in a set of *de novo* detected repeats.

Of the 35 genes with transposable element domain matches, 13 had other domains suggesting they could be host genes, and 28 overlapped spliced transcript alignments. Only four genes with either transposable element or unknown/uncharacterized domain family matches lacked evidence of being a host gene. That is these four genes had a single coding exon, lacked spliced transcript overlap and had no other InterPro domain match. We chose not to remove from the OGS genes that overlapped repeats detected by REPET or genes that matched InterPro transposable element domains, because of evidence supporting a large number of them as host genes, and the possibility that transposable elements may have contributed to the evolution of some host genes (e.g. reviewed in [44]).

## Discussion

In the approximately seven years that have elapsed between generation of OGSv1.0 and OGSv3.2, new sequence was added to the assembly; new sequencing technologies, genome assembly methods and gene prediction methods were applied; and new sources of gene prediction evidence became available. Several lines of evidence indicate that the new assembly is more complete. These include improved continuity of scaffolds, increased coverage of low GC content regions of the genome, identification of 782 new genes that do not align to the older assembly, and increased number of detectable repetitive elements. The upgraded assembly allowed us to conduct a comprehensive analysis of repetitive elements in the genome, create an improved gene set and confirm previous findings related to GC composition. It also allowed us to confirm that low amounts of transposable elements and repetitive DNA are bona fide features of the honey bee genome, and not artifacts of incomplete assembly and annotation.

Transposable elements can be a major factor in genome and gene evolution. Previous analyses found a low number of transposons and retrotransposons in the assembled genome compared to other sequenced insect genomes [1], but researchers questioned whether additional elements were present in unassembled portions of the genome. The upgraded assembly allowed us to better characterize repetitive elements in the genome. Despite a more comprehensive repetitive element annotation, the genomic coverage of transposable elements was extremely low, most striking for the retrotransposons, in agreement with the previous analyses. The most apparent difference between *A. mellifera* and other hymenopteran insects is the relative lack of retrotransposable elements; genomes of other bee, wasp, ant and insect species all contain higher proportions [22,23,45]. Although comparisons across studies are difficult due to methodological differences, our results show that the total fraction of repetitive DNA in the *A. mellifera* genome (9.46%) is lower than that of most other sequenced hymenoptera genomes. Some of the ant genomes have more than twice as much repetitive DNA (*Atta cephalotes*, 25%, *Linepithema humile* 23.5%, *Acromyrmex echinator* 27%, *Harpegnathos saltator* 27%, *Solenopsis invicta* >23%) and the genome of the parasitoid wasp, *Nasonia vitripennis*, contains more than three times the amount (>30%) [18-20,22-24]. The ants *Camponotus floridanus* and *Pogonomyrmex barbatus* are more similar to *A. mellifera*, with estimates of 15% and 9%, respectively [18,21]. However, those analyses did not include LARD, TRIM and MITE elements, which make up a considerable fraction of the repetitive elements in *A. mellifera* (4.1% of the genome; 43.3% of the repetitive DNA). Without these derivative elements, *A. mellifera* would possess well less than 1% transposable elements. This extraordinarily low proportion of mobile elements suggests that evolutionary processes molding the *A. mellifera* genome differ from processes working on other hymenopteran genomes, even though many of the species listed above are also insects with eusocial lifestyles.

Combined with new biological evidence for gene predictions, the upgraded assembly allowed for significant improvement to the *A. mellifera* gene set, with >50% more genes. The identification of 269 additional InterPro domains and the 57% reduction in number of missing universal insect orthologs indicate a more comprehensive catalog of protein functions. The presence of nine new venom protein genes, the detection of new tryptic peptides in existing venom protein genes, and the 53% increase in the number of computationally identified olfactory receptor domains are examples of more comprehensive annotation of specific gene families important to bee biology enabled by the improved genome.

The previously described *A. mellifera* genome characteristics of low and heterogeneous GC content [1] remain after the addition of new sequence to the assembly. Expansion and improvement of the low GC content regions in the new Amel_4.5 assembly was supported by the identification of Type I New genes, which were found in regions of lower GC content than that of Previously Known genes. While it is impossible to know whether new gene prediction evidence (transcript, peptide, homolog, genome conservation) affected the ability to predict the Type I New genes, the identification of Type II New genes in regions with slightly higher GC content than Previously Known genes suggested that the addition of new gene evidence had a greater impact on gene prediction in higher GC regions. Although any single evidence type was less frequent in the Type II New genes, the total number of Type II New genes supported by the evidence (3,555) was higher than that of Type I New genes (734). Higher recombination rates [46] and rates of molecular evolution in high GC content regions of the *A. mellifera* genome [47] may have contributed to sequence divergence that made Type II New genes difficult to detect when generating OGSv1.0. High GC content regions have been shown to be enriched in genes associated with behavioral traits [47], suggesting that some Type II New genes may be associated with important bee-specific traits.

Despite the higher mean GC content of regions containing Type II New genes, the strong bias for *A. mellifera* genes to reside in low GC content regions relative to the genome [1] remains. Among the wide range of insect genomes we have examined thus far, only the hymenopterans *A. mellifera* and *H. saltator* show a strong bias for genes to occur in compositional domains with low GC content, although *S. invicta*, *P. barbatus*, *L. humile* and *N. vitripennis* show a slight bias [48]. The biological meaning of this, and whether this is related to the lifestyles of these hymenopterans, is still unclear.

Comparisons of the old and new gene sets suggested that short and single coding exon genes, with spatially- or temporally-restricted expression patterns and low protein homology remain difficult genes to predict. Compared with Previously Known genes, Type II New genes were more rarely and narrowly expressed, had shorter coding sequences, were more likely to be single coding exon genes, and were less likely to have detectable homologs. The characteristics of Type II New genes were consistent with those of new genes identified in the modEncode effort to reannotate the developmental transcriptome of Drosophila melanogaster [49]. Their “new transcribed regions” (NTRs) in Drosophila also had low expression levels with temporally restricted patterns. In addition more than half of these NTRs were single-exon genes, and the multi-exon NTRs were shorter and less conserved than previously annotated genes [49]. While difficulty in computational identification of functional small open-reading frames (smORFs; <100 codons) has led to their low representation in genome annotations, increasing evidence supports functional smORFs in eukaryotes [50,51].

Efficient and effective annotation methods for non-model organisms has become critical in a time when initiatives such as i5K (5000 insect genomes [52]) and G10K (10,000 animal genomes [53]) motivate scientists to investigate genomes of diverse organisms, many of which will be evolutionarily distant from existing model organisms. Our results suggest that investigators wishing to comprehensively annotate protein-coding genes in genomes of non-model organisms should invest in transcriptome sequencing that is both deep and broad, similar to findings in re-annotation of the green anole lizard (*Anolis carolinensis*) genome [54]. Type II New genes were more likely to be narrowly expressed, so transcript evidence from only a single tissue may have missed a high proportion of the expressed genes. Transcriptome sequence from multiple tissues, life stages and conditions may be more useful than protein homolog evidence; the number of Type II New genes with transcript evidence exceeded the number of those with protein homolog evidence, despite the relatively close evolutionary distance between *A. mellifera* and some of the reference species, which included six species within the order Hymenoptera. Even with sampling many transcriptomes, rarely expressed genes can be missed, and genomic sequence from closely related species can aid gene prediction by leveraging nucleotide sequence conservation; our analysis showed that 752 of the OGSv3.2 genes were supported by genome conservation as their only source of biological evidence. As more genomes are sequenced and annotated, gene prediction should become easier. When expression and homolog evidence are lacking, it is important to train *ab initio* prediction algorithms using genes representing the true distribution of gene features such as coding sequence and exon length, codon usage and GC content. Our analysis of Type I and Type II New genes suggests that the bimodal distribution of genome GC content in *A. mellifera* results in at least two classes of genes distinguished by different distributions of GC content and codon usage. *Ab initio* gene predictors may benefit by training with coding sequences from each class separately.

## Conclusions

We have shown that next generation sequencing followed by *de novo* annotation can substantially improve an unfinished first generation genome sequence assembly and annotation. The upgraded assembly and annotation allowed us to confirm that the honey bee genome contains a typical number of genes relative to other insects. The improved honey bee gene set will be invaluable to the honey bee research community in efforts to elucidate the mechanisms behind fundamental biological processes, such as evolution of insect eusociality, as well as agricultural issues, such as pollinator health and immunity. Furthermore, understanding the reasons genes were not predicted in OGSv1.0 will lead to more effective gene prediction strategies for new genome projects.

## Methods

### Genome sequencing and assembly

We improved the published genome assembly of 2.7 million Sanger reads of the honey bee genome, version Amel_4.0 [1] by incorporating ABI Solid sequence and Roche 454 paired-end sequence to superscaffold and gap-fill the Amel_4.0 assembly using the Atlas-Link software [55]. The new sequence data as well as the existing sequence data were used to link the genome contigs from Amel_4.0 into more contiguous scaffolds. Adjacent contig sequences within these scaffolds were assessed and overlapping and redundant contigs were merged.

We used the paired-end reads for superscaffolding and intra-scaffold gap filling. The Amel_4.5 assembly contains new scaffolds formed from merging existing scaffolds and filling some intra-scaffold gaps with other scaffolds or contigs.

The newly formed scaffolds were anchored to the same linkage group that their member contigs were anchored to in Amel_4.0. Ten of the new scaffolds could be anchored to two different linkage groups so a manual break was inserted to split these scaffolds for consistent anchoring.

### New data for gene prediction

#### ***RNAseq data***

We sequenced samples from a number of tissues using the 454 Titanium technology. Tissues from ovary, testes, mixed antennae (worker, drones, and queens), larvae, mixed embryos, abdomen, and a combined library from brain and ovary samples were sequenced.

The testes cDNA library was prepared with the Clontech “full-length” amplification protocol. A gel cut of 400 to 800 bp fragments was combined with nebulized products from the larger cDNA fragments to generate fragments of an optimum sized sequencing library for the 454 Titanium platform. Other libraries were prepared by isolating total RNA using Trizol and RNeasy columns followed by mRNA isolation using the Qiagen kit and cDNA genration using the Invitrogen Superscript kit #11917-010 with random primers. A gel cut of 400 to 800 bp fragments was combined with nebulized products from the larger cDNA fragments to generate fragments of an optimum sized sequencing library for the 454 Titanium platform.

#### ***Peptide data***

##### 

**Honey Bee peptide atlas** We performed liquid chromatography-tandem mass spectrometric (LC-MS/MS) analysis of bee protein extracts from 253 samples, representing three castes, larvae and virtually all adult honey bee tissues in both sexes and of different disease states. Tissue collection and interpretation of the LC-MS/MS data has largely been described elsewhere [56-61] and is available for public download from the Honey Bee Peptide Atlas [62]. The raw data from these studies, amounting to more than 8 × 10^6^ tandem mass spectra, were searched against a six-frame translation (3,596,047 forward sequences, with reversed complemented sequences and common contaminants concatenated) of Amel_4.5 using Mascot (v2.3, Matrix Science). This exercise identified 30,622 unique peptide sequences at a false discovery rate of 1%, of which 5,834 peptides mapped to regions of the genome, where genes were previously unknown in Amel_4.0. As these results were based solely on a basic translation of raw genomic sequence, these peptides represent only tryptic peptides wholly within a single exon.

##### 

**Venom peptides** We analyzed the honey bee worker venom proteome by integrating a combinatorial peptide ligand library (CPLL) with liquid chromatography/Fourier transform ion cyclotron resonance (LC-FTICR) MS/MS. The collection and interpretation of the MS/MS data are described elsewhere [35]. Gene prediction datasets and a six-frame translation of Amel_4.5 were searched using Mascot (v2.3, Matrix Science). Setting the significance threshold at p < 0.01 led to a peptide false discovery rate of 5.23% for the search of the AUGUSTUS AU9 gene set, 2.51% for the AUGUSTUS AU11 search and 3.77% for the Amel_4.5 NCBI RefSeq search. MS/MS data generated from the CPLL flow-through fractions, and the elution fractions separated by Tris-glycine- or Tris-tricine-SDS-PAGE gels were separately searched against the genome six-frame translation resulting in false discovery rates of respectively 1.56%, 3.75% and 3.44%.

### Annotation methods

#### ***Summary of gene prediction sets***

Genes were predicted using a variety of methods including the NCBI RefSeq and Gnomon pipelines, AUGUSTUS, SGP2, GeneID, Fgenesh++ and N-SCAN. Unless stated otherwise below, gene prediction pipelines used the masked assembly available from NCBI [63], which was generated using RepeatMasker [64]. New transcriptome data was used either directly as evidence or in the generation of training sets for the predictors. The Augustus analysis also incorporated available peptide data, and the N-SCAN analysis leveraged nucleotide sequence conservation between the *A. mellifera* genome and the other bee genomes. SGP2 leveraged conservation between the *A. mellifera* genome and the previously published *Nasonia* genomes [23] based on translated alignments.

#### ***NCBI RefSeq and Gnomon***

The Amel_4.5 assembly was annotated with NCBI's eukaryotic genome annotation pipeline (version 3.0), as described at [65]. The NCBI pipeline uses a repeat-masked assembly generated by WindowsMasker [66] and transcript and protein alignment evidence supplemented with *ab initio* prediction to annotate coding and non-coding transcripts and proteins. The evidence used for this annotation run included alignments of:

1) 9 million RNAseq reads from 454 sequencing (described above), which were treated as ESTs in the pipeline.

2) All available honey bee ESTs and mRNAs.

3) Proteins from the FlyBase annotation of *Drosophila melanogaster* (release 5.30).

4) Proteins from the RefSeq annotation of human.

5) Proteins annotated on insect mRNAs.

6) Proteins from the annotations of the ant genomes, *Harpegnathos saltator* and *Camponotus floridanus* available in GenBank.

The final RefSeq annotation included models that were completely or partially supported by alignment evidence. The pipeline had some provisions to predict models that were disrupted by frameshifts or stop codons in the assembly, some of which were assigned protein accessions (XP_ prefixes) and named with the prefix ‘LOW QUALITY PROTEIN’, whereas others were conservatively classified as pseudogenes and not assigned protein accessions. The RefSeq annotation is available from NCBI's genomes FTP site as NCBI build 5.1 [63].

#### ***AUGUSTUS***

AUGUSTUS can be used as an *ab initio* gene prediction tool, but can also integrate extrinsic evidence from various sources [67].

#### ***Generation of training gene structures and training AUGUSTUS***

We used Scipio [68] to generate gene structures on the Amel_4.5 assembly with a protein set from a previous *A. mellifera* annotation (Amel_pre_release2_OGS_pep.fa). We used these gene structures to optimize AUGUSTUS parameters for *A. mellifera*, and constructed UTR models from 78,274 *A. mellifera* ESTs from GenBank, which were used to train AUGUSTUS UTR parameters. We then predicted genes in the Amel_4.5 assembly. Individual RNAseq reads from 454 sequencing (described above) were mapped against predicted transcripts, and fully covered transcripts (2,151) were selected as training genes for optimizing a final AUGUSTUS parameter set.

#### ***Gene prediction with AUGUSTUS***

We generated three gene sets using AUGUSTUS: a gene set with extrinsic evidence from ESTs and RNAseq data (AU9), a particularly inclusive gene set that contained many alternative transcripts for peptide identification (AU11), and a gene set with extrinsic evidence from ESTs, RNAseq data and peptide data (AU12) (Table S6 in Additional file 1).

We created extrinsic evidence “hints” for protein coding genes and transcripts from the *A. mellifera* ESTs and from 454 transcriptome libraries. Genes were predicted with AUGUSTUS, allowing the prediction of alternative transcripts and allowing the splice site AT-AC (in addition to GT-AG and GC-AG) in case of supporting extrinsic evidence. The resulting gene set was named AU9. AU9 genes were predicted using the following options: 2augustus –species = honeybee1 –UTR = on –print_utr = on –hintsfile = all_but_no_peptide.hints –extrinsicCfgFile = extrinsic.M.RM.E.W.cfg –exonnames = on –codingseq = on –alternatives-from-evidence = true –allow_hinted_splicesites = atac genome.fa

For the purpose of peptide identification, alternative transcripts were extensively sampled with AUGUSTUS, resulting in the gene set AU11. AU11 genes were predicted issuing the following command: augustus –UTR = on –print_utr = on –hintsfile = all_but_no_peptide.hints –extrinsicCfgFile = extrinsic.M.RM.E.W.cfg –exonnames = on –codingseq = on –species = honeybee1 –alternatives-from-evidence = true –alternatives-from-sampling = true –sample = 100 –minexonintronprob = 0.1 –minmeanexonintronprob = 0.4 –maxtracks = -1 –allow_hinted_splicesites = atac genome.fa

We generated hints from peptide sequences by mapping the peptides against the protein set of AU11 and against a six-frame translation of the genome using BLAT [69] in a non-redundant way (i.e. parts of the six-frame translation that were included in the protein set AU11 and redundant parts of the AU11 protein set that were removed). A final AUGUSTUS gene set AU12 was generated using all available extrinsic evidence and the following options: augustus –species = honeybee1 –UTR = on –print_utr = on –hintsfile = all.hints –extrinsicCfgFile = extrinsic.M.RM.E.W.cfg –exonnames = on –codingseq = on –alternatives-from-evidence = true –allow_hinted_splicesites = atac genome.fa.

#### ***Fgenesh++***

Predictions were made using FGENESH 3.1.1 [70,71] using the HBEE matrix with parameters specific for *A. mellifera*. We used Illumina transcriptome data from *A. mellifera* forager and nurse brains available from the SRA (SRP003528), which were a total of 181.8 M spots, 18.3G bases of 100 bp single end reads generated on an Illumina Genome Analyzer II in 2010. We mapped the Illumina RNASeq using the ReadsMap program [72], which provided intron position information to Fgenesh for building sample-specific gene models. Predictions were made based on individuals used in Illumina RNASeq libraries (five nurses and five foragers), and then were combined into forager and nurse group predictions, and redundant genes (those having coinciding coding sequences) were removed from each set. Finally, the forager and nurse predictions were combined into a final set of predictions, and redundant genes (those having coinciding coding sequences) were removed from this combined set to produce the final Fgenesh++ prediction set.

#### ***GeneID***

GeneID is an *ab initio* gene prediction program used to find potential protein-coding genes in anonymous genomic sequences. The training of GeneID to obtain a parameter file for *A. mellifera* was based on the method described to obtain a *Drosophila melanogaster* GeneID parameter file [73]. Training was performed in a “semi-automated” manner by employing a recently developed GeneID training tool that computes position weight matrices (PWMs) or Markov models of order 1 for splice sites and start codons, and derives a model of coding DNA, which, in this case, is a Markov model of order 5. Furthermore, once a preliminary species-specific matrix is obtained it is further optimized by adjusting two internal matrix parameters: -the cutoff of the scores of the predicted exons (eWF) and the ratio of signal to coding statistics information to be used (oWF).

The initial *A. mellifera* training set was comprised of the 2,151 gene models used to train AUGUSTUS (described above). Of these gene models 80% (1,720) were used to train GeneID while the remaining 20% (431) were set aside to test the accuracy of the newly developed matrix. The 1,720 *A. mellifera* protein-coding gene models included 7,913 canonical donor splice sites/7,939 canonical acceptor sites and 1,720 start codons. The start codons were used to compute PWMs while the donor and acceptors were used to derive Markov matrices of order 1. Given the large number of sequences we also had enough coding (2,338,800) and non-coding (5,561,154) nucleotides to derive a Markov of order 5 for the coding potential. We tested accuracy of the GeneID *A. mellifera* parameter file on an artificial contig consisting of the 431 evaluation-set concatenated gene models with 800 nucleotides of intervening sequence between each of the genes (Table S7 in Additional file 1). We then used the GeneID parameter files to predict genes on an assembly consisting of Amel_4.5 sixteen chromosomes and 5,304 "unplaced" scaffolds files that had repeat sequences masked using Repeatmasker. GeneID predicted 24,554 protein-coding genes.

#### ***SGP2***

SGP2 is a syntenic gene prediction tool that combines *ab initio* gene prediction (GeneID) with TBLASTX searches between two or more genome sequences to provide both sensitive and specific gene predictions, and it tends to improve GeneID’s performance, especially by reducing the number of false-positive predictions. SGP2 requires one or more reference genomes to which the target genome (in this case *A. mellifera*) is compared. We decided to use the genomes of *Nasonia vitripennis*, *N. giraulti* and *N. longicornis*[23] as references to develop our *A. mellifera* parameter file for SGP2 because the genus *Nasonia* is at an appropriate evolutionary distance from *Apis* such that mostly the coding regions of the genes, not the introns or intergenic regions, are significantly conserved between these two genomes.

Obtaining the SGP2 *A. mellifera*-specific parameter file was based on the methodology described by Parra *et al*[74] used to obtain a human SGP2 parameter file using mouse homology evidence. The starting point to obtain a parameter file for SGP2 was the previously described GeneID *A. mellifera* matrix. The GeneID-derived SGP2 parameter file was optimized on an artificial contig comprising the same concatenated 1,720 sequences used to train GeneID, with 800 nucleotides between each of the gene models. We optimized the SGP2 matrix by modifying not only the eWF internal parameter (as previously for the GeneID parameter file) but also two SGP2-specific internal parameters (“NO_SCORE” and “HSP_factor”). The “NO_SCORE” parameter provides a penalty for no overlap between TBLASTX-derived HSPs (High-Scoring Pairs) and GeneID *ab initio* predictions in the same region whereas the “HSP_factor” parameter reduces the score assigned to the HSPs in order to maximize the prediction accuracy. We evaluated the newly developed SGP2 parameter file on the same artificial contig consisting of the 431 concatenated gene models with 800 nucleotides of intervening sequence between each of the genes used to evaluate the GeneID bee matrix (Table 1). We then used the SGP2 parameter file2 to predict genes on the same assembly file used for GeneID, and generated 20,179 predictions.

#### ***N-SCAN***

We used the N-SCAN package [75] to leverage conservation between the *A. mellifera* genome and genomes of two other bee species, *A. florea* (Aflo_1.0 ) and *Bombus terrestris* (Bter_1.0). We first masked Amel_4.5 for simple sequence repeats using RepeatMasker [64]. We ran LASTZ [76] using default parameters with Amel_4.5 as the target genome, and either *Apis florea* (Aflo_1.0) or *Bombus terrestris* (Bter_1.0) as the informant genome. We then used iParameterEstimation to generate both an Amel_4.5-Aflo_1.0 specific parameter set as well as an Amel_4.5-Bter_1.0 parameter set using the training set described above for AUGUSTUS gene prediction, including UTR features. Finally, we ran N-SCAN using each of the *A. mellilfera* specific parameter sets with the respective LASTZ informant alignments to produce two N-SCAN prediction sets, one set based on Aflo_1.0 as the informant genome and the other set based on Bter_1.0 as the informant genome.

### Combining gene sets

#### ***Input data for combined gene sets***

We used MAKER2 and GLEAN to generate combined gene sets. The MAKER2 and GLEAN analyses used the same set of input data. Both analyses combined the gene predictions described above (NCBI, AUGUSTUS, Fgenesh++ with RNAseq, N-SCAN using *A. florea* as an informant genome, GeneID and SGP2) with transcript and protein homolog alignments. Transcript data included the new 454 transcriptome data described above, *A. mellifera* ESTs from GenBank and Illumina nurse and forager reads downloaded from the SRA (SRP003528, described above).

We aligned Illumina reads to Amel_4.5 in two groups, nurse and forager, using Tophat version 1.3.1 with the option "--butterfly-search" for more sensitive splice junction detection, and then generated predicted transcripts for each set of pooled data using Cufflinks version 1.0.3 with default parameters. The 454 reads were assembled into contigs *de novo* using Newbler (2.3-PreRelease-9/14/2009) with the cDNA option. We aligned 454 contigs and ESTs to Amel_4.5 using MAKER2 v2.15, which uses WU-BLAST [77] and Exonerate est2genome [78], with minimum 80% alignment coverage and 95% identity. Protein homolog alignments included SwissProt [79] Metazoa homologs, *Drosophila melanogaster* (fruit fly; r5.31) [80], *Nasonia vitripennis* (parasitoid wasp; OGSv1.2) [23] and the ants: *Atta cephalotes* (OGSv1.1) [22], *Camponotus floridanus* (OGSv3.3) [18], *Harpegnathos saltator* (OGSv3.3) [18], *Linepithema humile* (OGSv1.1) [20], *Pogonomyrmex barbatus* (OGSv1.1) [21]). Proteins in the SwissProt dataset annotated as transposable elements were removed prior to alignment. We aligned protein sequences to Amel_4.5 using Exonerate protein2genome with a minimum 60% percent identity and 60% alignment coverage.

#### ***MAKER2***

To create a combined gene set, we ran MAKER2 v2.15 using parameters min_contig = 1000 and pred_gff, which allowed us to provide as input the gene prediction sets and alignments described above, instead of generating new evidence tracks within MAKER2.

#### ***GLEAN***

We ran GLEAN [17] 32 times to create consensus gene sets using different combinations of the gene prediction sets described above. All of the GLEAN runs included the transcript and protein homolog alignments described above, and required a minimum coding sequence length of 75 nt.

#### ***Selecting the new official gene Set***

We evaluated the 32 GLEAN sets based on several criteria including overlap with a conservative evidence-based set (RefSeq), transcript sequences, peptides and the CEGMA conserved core set [30] (Additional file 2). NCBI’s RefSeq pipeline has been considered reliable and relatively conservative, so we performed overlap analyses to determine how many of the RefSeq models were captured in the GLEAN consensus sets. We used FASTA [81] to align GLEAN coding sequences with RefSeq coding sequences where a perfect alignment required 99% identity over the entire lengths of both sequences. We used FASTA to align peptides to GLEAN proteins, with E-value and scoring matrix optimized for short exact matches (E-value .01 and MD10 scoring matrix). We parsed peptide alignments to count those with 100% identity and 100% alignment coverage (Additional file 2).

In addition to alignments between GLEAN predictions and RefSeq genes and peptides, we considered numbers of gene models, total numbers of coding nucleotides, average coding sequence lengths, and numbers of splits and merges compared to RefSeq. We selected the GLEAN31 set (Additional file 2) to be the official gene set, OGSv3.2. It was the set generated using gene predictions from NCBI Gnomon, AUGUSTUS, Fgenesh++, N-SCAN using *A. florea* as an informant genome and GeneID and SGP2 combined as a single set as well as the transcript and protein homolog alignments.

#### ***Adding UTRs to OGSv3.2 gene models***

Although we did not use MAKER2 to generate the official gene set, we did use MAKER2 (v2.15) [28] to add UTR to the GLEAN coding exons since GLEAN does not produce annotations with UTR features. OGSv3.2 coding exons were input as “pred_gff” and transcriptome evidence (including the 454 transcripts, Illumina contigs and *A. mellifera* ESTs described above) was input as “est”. MAKER2 aligned the EST evidence to Amel_4.5 using WU-BLAST and Exonerate est2genome then used overlapping EST evidence to extend OGSv3.2 models to include UTR features when possible. Because MAKER2 sometimes modified the coding exon coordinates, we processed the MAKER2 output and retained UTRs only when the coding exon structure was unchanged. Out of a total of 15,314 OGSv3.2 genes, UTR were added to 7,514 genes (49%).

### Identifying and characterizing of new genes

#### ***Identifying of new and previously known OGSv3.2 genes***

In order to compare the newest gene set (OGSv3.2) with the first official gene set (OGSv1.0), we mapped OGSv3.2 coding sequences to Amel_2.0, which was the assembly on which OGSv1.0 was generated [1]. First, we used MegaBLAST [82] to identify scaffold/gene matches with 95% identity and E-value < 1 × 10^-20^. We then aligned coding sequences to matching scaffolds using GMAP [83], and parsed the output to create two sets of splice-modeled alignments, both requiring 95% identity. One set was based on a relaxed coverage criterion, requiring that the alignment cover at least 50% of the coding sequence. The other set was based on a stringent coverage criterion, requiring that the alignment cover 80% of the coding sequence. Results of further analyses for the relaxed mapping set are provided in the supplemental materials, but we discuss only results for the stringent mapping.

On the basis of mapping OGSv3.2 to Amel_2.0 and overlap between OGSv3.2 and OGSv1.0 gene models on the Amel_2.0 assembly, we divided 15,314 OGSv3.2 genes into three sub-sets (Table 6). We deemed any OGSv3.2 gene that did not align to the Amel_2.0 assembly a “Type I New” gene. The additional sequencing and reassembly of the genome for the Amel_4.5 assembly likely allowed the detection of these genes. “Type II New” genes were those that did align to the Amel_2.0 assembly, but whose coordinates did not overlap an OGSv1.0 gene by a single coding base pair on the same strand. Additional expressed sequence and protein homolog evidence as well as improvements to gene prediction algorithms likely contributed to the detection of these genes. Finally, any OGSv3.2 gene that both aligned to the Amel_2.0 assembly and overlapped an OGSv1.0 gene was deemed a “Previously Known” gene.

#### ***Coding sequence length analysis***

The total length of the coding sequence was calculated for each gene and the means for all genes and each gene sub-set were calculated (Table 6). We tested the null hypotheses that the mean coding sequence lengths of Type I and Type II New genes and Previously Known genes were equal.

#### ***Splice site and single versus multiple coding exon gene analysis***

We assessed the genomic sequence of the two intronic base pairs adjacent to each coding sequence exon-intron splice site to determine whether they corresponded to the canonical …]5’-GT/AG-3’[… splice site sequence. We considered only splice sites supported by matching intron coordinates of spliced transcript alignment evidence. We used chi-square tests with one degree of freedom to compare the frequencies of single coding exon genes and non-canonical splice sites in Type I or Type II New genes with Previously Known genes.

#### ***OGSv3.2 gene location relative to GC compositional domains***

We used IsoPlotter, a recursive segmentation algorithm [84,85], to partition the *A. mellifera* genome into GC compositionally homogeneous domains, contiguous regions of the genome with similar GC content (percent G + C nucleotides). We determined the GC content of the GC compositional domain for each OGSv3.2 gene. In cases where a gene spanned multiple GC compositional domains, we calculated the average GC content of the GC compositional domains, and weighted it according to the fraction of the gene's length that occurs in each GC compositional domain. We computed the weighted percent GC based only on non-coding nucleotides of the compositional domains, because we did not wish to include effects of codon bias.

We compared the distribution of Type I and Type II New genes with respect to GC content to that of Previously Known genes. IsoPlotter cannot segment scaffolds less than 10 kb into compositional domains, so the 90 genes residing in these short scaffolds were not considered. We tested the null hypotheses that the means of the weighted GC compositional domain contents for genes in the Type I and Type II New gene sets were equal to the mean of the Previously Known genes.

#### ***Effective number of codons analysis***

Using the chips program within the EMBOSS package [86], we calculated the effective number of codons separately for each OGSv3.2 gene. We tested the null hypotheses that the mean effective number of codons values for the Type I and Type II New genes were equal to the mean of the Previously Known genes.

#### ***Tissue expression analysis***

We determined the number of OGSv3.2 gene overlaps to transcript alignments that were used in creating the GLEAN consensus gene sets (454 reads, Illumina contigs and *A. mellifera* ESTs from GenBank, described above). We relied on splice signals to determine the directionality of a transcript read. We tallied spliced and unspliced alignments separately, since we could be confident when directionality of a spliced alignment agreed with a gene prediction, but could not be confident about unspliced alignments. For spliced transcript alignments, if the transcript was on the opposite strand from the gene then it was discarded from further analysis. For transcripts on the same strand or transcripts that were un-spliced, in which case directionality could not be determined, a coordinate overlap of at least one coding base pair was required for a gene to count as overlapping with a transcript alignment. We counted the number of transcript data sets in which each OGSv3.2 gene was found to have an overlapping alignment (Table 6.) We performed chi-square tests with one degree of freedom to compare the frequencies of spliced transcript overlap in the Type I or Type II New gene sets with the Previously Known gene set.

Of genes that overlapped spliced transcript alignments, we identified genes that were narrowly expressed and genes that were broadly expressed on the basis of overlap to the four single-tissue libraries (brain [combined Illumina forager and nurse brain libraries] and 454 libraries of mixed antennae, ovary and testes). (Foragers and nurses are worker honey bees that specialize on collecting food and feeding brood, respectively.) Genes were deemed narrowly expressed if they overlapped at least one transcript alignment in only one of the four tissues and broadly expressed if they overlapped at least one transcript alignment from all four tissues. We performed chi-square tests with one degree of freedom to compare the frequencies of narrowly expressed genes and broadly expressed genes in the Type I or Type II New gene sets with the Previously Known gene set.

#### ***Homolog analysis***

We determined the number of protein alignments overlapping OGSv3.2 for protein data sets that were used in creating the GLEAN consensus gene sets. We required overlap of at least one coding base pair on the same strand to deem a gene overlapping with a protein homolog alignment. We performed chi-square tests with one degree of freedom to compare the frequency of the existence of overlaps to homolog alignments for Type I or Type II New genes with that of Previously Known genes.

#### ***TBLASTN alignment of OGSv3.2 to A. florea and B. terrestris genomes***

We aligned OGSv3.2 protein sequences to the *A. florea* (Aflo_1.0) and *B. terrestris* (Bter_1.0) genome assemblies using TBLASTN [87] with an E-value criteria of 1 × 10^-06^. We did not use information about predicted genes or expression in *A. florea* or *B. terrestris*.

#### ***Statistical methods to test for differences in means between new and previously known genes***

To test the null hypothesis that the mean of a particular variable for Type I or Type II New genes was equal to the mean of the Previously Known genes, we used both a non-parametric Kolmogorov-Smirnov test and a Welch t-test with the correction for non-homogeneity of variances. Testing the hypotheses in this way avoids assuming these data were normally distributed or had equal variances. To be conservative, we report the least significant P-value for each test.

### Using peptide data in development and analysis of gene sets

We used the peptide data is described above to evaluate the GLEAN sets and analyze Type I New, Type II New, and Previously Known genes. We aligned the peptide sequences to predicted protein sequences with FASTA [81] with a relaxed E-value of 0.1 and the MD10 scoring matrix. These parameters were found to allow matches to peptide sequences as short as 6 amino acids with 100% identity. Only alignments with 100% identity were retained. We used chi-square tests with one degree of freedom to compare the frequency of proteins that aligned to peptide sequences in Type I or Type II New genes with that of Previously Known genes.

The venom peptide data were used separately to evaluate gene sets for improved identification of venom genes. All significant and top ranking venom peptides from the Mascot output, with an ion score ≥30 were retained in the final peptide lists. Venom mass spectra were searched against the OGSv3.2 and OGSv1.0 to compare the contribution of both datasets to the identification of new venom genes. The false discovery rates of both searches were set to 1%.

Venom peptide data were used to annotate venom genes using the Apollo annotation tool [36], provided for Amel_4.5 by the Hymenoptera Genome Database [88].

### Assessing orthology

The protein-coding gene annotations from the two *A. mellifera* genome assemblies were compared with orthologs from OrthoDB [89] from nine other insects. These included *Pediculus humanus* (PhumU1.2, 10,772 genes), *Acyrthosiphon pisum* (ACYPI v2.1, 36,275), *Nasonia vitripennis* (nvit2, 24,369), *Linepithema humile* (OGSv1.2, 16,048), *Pogonomyrmex barbatus* (OGSv1.2, 17,100), *Tribolium castaneum* (Tcas 3.0, 16,565), *Danaus plexippus* (OGS2, 15,329), *Anopheles gambiae* (AgamP3.6, 12,670), and *Drosophila melanagaster* (r5.45, 13,927). For annotations on the older *A. mellifera* genome assembly, we used the gene set known to the community as “Amel_prerelease2” (abbreviated as V2), available from BeeBase. It was the gene set resulting from mapping OGSv1.0 from assembly Amel_2.0 to assembly Amel_4.0, and included a small number of manual annotations, with a total of 10,699 genes. Comparing each *Apis* annotation (V2 and OGSv3.2) to the other nine gene sets identified near-universal orthologous groups with orthologs in all but one or all but two insects. For each gene set, Figure 4 and Table S5 in Additional file 1 show counts of near-universal insect orthologous groups that are missing orthologs in different species. Total counts were partitioned into groups with only single-copy orthologs and those with gene duplications, further divided into those with only one missing species and those with two missing species.

### Predicting protein functions

#### ***GO analysis***

We used FASTA [81] with an E-value threshold of 1 × 10^-6^ to compute reciprocal alignments between OGSv3.2 proteins and a *D. melanogaster* protein set consisting of the longest protein isoform of each gene (annotation version r5.42). We identified reciprocal best hits (RBH) and transferred Gene Ontology (GO) [90] annotations from the *D. melanogaster* protein to the *A. mellifera* protein for each RBH pair, using the GO annotation file available at FlyBase [80]. We used GeneMerge [91] to test for enrichment of GO terms in OGSv3.2 protein datasets, testing for each of the three GO ontologies (Molecular Function, Biological Process and Cellular Component) separately. Several tests were performed with either the entire set of Gene Ontology terms, the generic GO slim set, or the GO slim set developed for *A. mellifera* by Whitfield et al. [92]. Population and test gene datasets for a particular GO ontology included only OGSv3.2 genes with GO annotations for that ontology. Population datasets consisted of all OGSv3.2 genes with GO annotations. Test datasets were 1) all new genes based on stringent mapping criteria, 2) Type I New genes using stringent criteria, 3) Type II New genes based on stringent mapping criteria.

#### ***InterPro analysis***

We used InterProScan [93] to compare OGSv3.2 and OGSv1.0 proteins with the following InterPro [29] protein domain and motif databases: PFAM [94], TIGRFAMS [95], SMART [96], PRODOM [97], PROSITE [98], PIRSF [99], GENE3D [100], SUPERFAMILY [101], and PANTHER [102]. The total numbers of proteins annotated with at least one InterPro domain were 9,479 and 8,552 for OGSv3.2 and OGSv1.0, respectively. For each InterPro domain identified in the combined datasets, we determined the number of proteins containing that domain within OGSv3.2 and OGSv1.0. Then for each InterPro domain, we used 2 × 2 chi-square tests with Yates correction and one degree of freedom to determine whether the frequencies of proteins containing that domain differed between the OGSv3.2 and OGSv1.0 InterPro-annotated sets (total 9,479 and 8,552 proteins, respectively).

### Detecting genome-wide repetitive elements

We detected and annotated repetitive elements with the REPET software package ([103], version 2.0) consisting of two pipelines integrating a set of bioinformatics programs. First, repeated sequences were detected by similarity, using an all-by-all BLAST [104] search via BLASTER [105]. LTR-retrotransposons were detected by structural search with LTRharvest [106]. The similarity matches were clustered with GROUPER [105], RECON [107] and PILER [108], the structural matches were clustered with NCBI BLASTclust [109]. From each cluster a consensus sequence was generated by multiple alignment with Map. We analyzed the consensus sequences for terminal repeats (TRsearch), tandem repeats (TRF), open reading frames (dbORF.py, REPET) and poly-A tails (polyAtail, REPET). We screened the consensuses for matches to nucleotide and amino acid sequences from known transposable elements (RepBase 17.01, [42]) using BLASTER [105], which runs tblastx and blastx [87], and searched them for HMM profiles (Pfam database 26.0, [94]) using HMMER3 [110]. Based on the detected structural features and homologies, we classified the consensuses using PASTEC according to Wicker et al. [111]. We then removed redundancies identified with BLASTER and MATCHER [105] as well as elements classified as simple sequence repeats (SSRs; >0.75 SSR coverage) or unclassified elements built from less than 10 fragments.

We used the set of *de novo* detected repetitive elements to mine the genome in a second pipeline with BLASTER (using NCBI BLAST, sensitivity 4, followed by MATCHER), RepeatMasker (using CrossMatch, sensitivity q, cutoff at 200) and CENSOR (using NCBI BLAST). We removed false positive matches using an empirical statistical filter. Satellite repeats were detected with TRF [112], MREPS [113]and RepeatMasker [64] and were then merged into a single set. We also screened the genomic sequences for matching nucleotide and amino acid sequences from known transposable elements (RepBase 17.01, [42]) with BLASTER. Finally we removed TE doublons (loci annotated as multiple transposable elements) and SSR annotations within transposable element annotations, and performed a "long join procedure" to connect distant fragments. Sequences from the *de novo* repetitive element library, which were found to have at least one perfect match in the genome were then used to rerun the whole analysis.

To ensure compatibility and to avoid introducing a bias, we refrained from a manual curation or clustering of the *de novo* detected elements before mining the genome. However, post hoc we manually analyzed all elements, which were previously classified into class I retrotransposon or class II DNA transposon elements or unclassified elements with detected coding element features (similarity to known transposable elements) due to potential chimeric insertion. At this stage we excluded derivative elements (LARD, TRIM, MITE) from further detailed inspection unless carrying a class I or class II element. Some elements were classified “potential Hostgene” in the computational analysis, based on characteristics of the DNA, not based on overlap analysis with predicted genes. These “potential Hostgene” elements, as well as unclassified elements (“noCat”), were also excluded from manual analysis. We performed manual inspection by checking for open-reading frames (ORF) with the NCBI ORF Finder (NCBI), by searching the NCBI Conserved Domain Database (CDD) [114], by searching the most up to date online RepBase database (accessed December 2012-February 2013) via CENSOR [115]. We also performed phylogenetic analysis for LINE RT domains with RTclass1 [116] in order to achieve a detailed classification for each element, determine its potential relation to a family of known elements, to evaluate the completeness and to detect potential active elements. We defined an element to be complete, if it possessed the relevant coding parts with the element-typical domains and the structural features (LTR, TIR). The potential activity was defined according to the region an intact ORF, if present, covered. If an intact ORF seemed to cover a complete region including the typical domains (e.g. GAG as well as POL, Tase) then the element was considered to be potentially active. If a Tase domain was covered by a truncated ORF or the Tase itself appeared to be truncated but was covered by an intact ORF, or if the RT domain was covered by an active ORF but not the remaining element-typical domains, then the element was considered to be maybe potentially active. During the manual classification to at least superfamily level, novel transposable element types not covered by the system of Wicker et al. [111] were also considered: Kolobok, Sola, Chapaev, Ginger, Academ, Novosib and ISL2EU class II DNA transposons [117,118].

Simple sequence repeats and other low complexity regions were extracted from the REPET pipeline database and processed with a custom Perl script to calculate the total coverage of these types of repetitive DNA by omitting overlaps with transposable element or other repetitive element annotations.

### Ethical approval

Experimental research followed appropriate guidelines for the ethical treatment of research subjects. Human subject and animal protocol approvals were not applicable.

### Data availability

The 454 transcript read data are available in the Sequence Read Archive (SRA) [119] at the NCBI (SRP003261, SRP003260, SRP001899). The assembled 454 transcripts (119,959) are in the Transcriptome Shotgun Assembly (TSA) database, and available through NCBI BioProject pages for PRJNA51481 and PRJNA51483 [120].

The accessions for the assembled 454 transcript data are: HP542035 to HP552088 (testes), HP527956 to HP542034 (mixed antennae), HP509343 to HP527955 (embryo), HP482918 to HP509342 (brain; ovary), HP473811 to HP482917 (larvae), HP459439 to HP473810 (abdomen), and HP552089 to HP579397 (ovary).

The SOLID genomic read data are available in the SRA (SRX097020). The 454 genomic read data are also available (SRX006752, SRX000071).

The Amel_4.5 assembly is available from NCBI under the accession GCA_000002195.1.

The following resources are available at BeeBase [121], a division of the Hymenoptera Genome Database [88]: genome browsers with gene annotations and supporting evidence alignments; BLAST databases with the Amel_4.5 scaffold assembly, OGSv3.2, all input gene prediction sets and transcript contig assemblies; and a data download page with fasta sequence files and gff for annotations. Peptides and their tandem mass spectra are available from the *Apis mellifera* PeptideAtlas [62].

## Abbreviations

BAC: Bacterial artificial chromosome; CPLL: Combinatorial peptide ligand library; DIRS: Dictyostelium intermediate repeat sequence; ENC: Effective number of codons; EST: Expressed sequence tag; FTICR: Fourier transform ion cyclotron resonance; GAG: GAG-Protein of retrotransposons; GO: Gene Ontology; HMM: Hidden Markov model; HSP: High scoring pair; LARD: Large retrotransposon derivative; LC: Liquid chromatography; LINE: Long interspersed element; LTR: Long terminal repeat; MITE: Miniatiure inverted-repeat transposable element; MS/MS: Tandem mass spectrometry; nt: Nucleotides; NTR: New transcribed region; OGS: Official gene set; ORF: Open reading frame; POL: POL-polyprotein of retrotransposons; PWM: Position weight matrix; RBH: Reciprocal best hit; RT: Reverse transcriptase; SINE: Short interspersed element; SRA: Sequence Read Archive; SSR: Simple sequence repeat; TASE: Transposase; TE: Transposable element; TIR: Terminal inverted repeat; TRIM: Terminal repeat retrotransposon in miniature; UTR: Untranslated region.

## Competing interests

The authors declare that they have no competing interests.

## Authors’ contributions

Manuscript: CGE^1, 2^, KCW^3^, AKB^2^. Human Genome Sequencing Center Director: RAG^3^. Honey Bee Genome Phase II White Paper Authors: JDE^10^, MB^4^, CGE^1, 2^, RM^20^, HMR^24^, GER^25^, DBW^30^, CWW^31^. Sequence production: YW^3^ (cDNA sequencing libraries at BCM-HGSC), IFN^3^ (cDNA sequencing libraries at BCM-HGSC), CLK^3^, MH^3^, VJ^3^, DMM^3^, HGSC production teams^3^, GER^25^ (cDNA sequencing at U. Illinois). cDNA: RM^20^ (Brain & Ovary, Testes, Mixed Antennae, Embryos, Larvae), GER^25^ (Queen Ovary, Nurse, Forager), MEH^14^ (Nurse and Forager Brain), RSK^17^ (Nurse and Forager Brain), JDE^10^ (Abdomen), JD^3^ (transcript analysis and assembly), DZ^3^ (transcript analysis). Genome Assembly: KCW^3^, LZ^3^, HJ^16^, JD^3^. Genetic Map: GJH^15^, JMT^28^, JM^19^, OR^26^, ES^21^. Gene Annotation: CGE^1, 2^ (N-Scan, MAKER, GLEAN), AKB^2^ (N-Scan, MAKER), TDM^23^ (RefSeq, Gnomon), FC^5^ (SGP2, GeneID), RG^5^ (SGP2, GeneID), KJH^13^ (AUGUSTUS), PK^18^ (Fgenesh++), VS^27^ (Fgenesh++), MS^13^ (AUGUSTUS). OGS Evaluation and Characterization: CGE^1, 2^, AKB^2^. GC Composition: CGE^1, 2^, AKB^2^, EE^9^, DG^12^, JTR^2, 6^. Orthology Assessment: RMW^29^, EMZ^29^. Protein Functional Analysis (GO and InterPro): CGE^1, 2^. Genomic Repetitive Elements: ES^21^, RFAM^21^. Peptide data and analysis: MVV^7^ (venom peptides), LJF^11^ (PeptideAtlas), GD^8^ (venom peptides), BD^8^ (venom peptides), DCdG^7^ (venom peptides). Genome Browser, BLAST Website and Manual Annotation Software: CGE^1, 2^, AKB^2^, CPC^2, 6^, MCM-T^2, 22^, JTR^2, 6^. All authors read and approved the final manuscript.

## Supplementary Material

Additional file 1 **Is a document containing Tables S1 through S7 and Figure S1. Table S1.** provides details for genomic sequencing runs, listed by SRA run number. **Table S2.** provides a comparison of New and Previously Known OGSv3.2 genes based on relaxed mapping criteria. **Table S3.** provides a comparison of frequencies of canonical and non-canonical splice sites in New and Previously Known genes. **Table S4.** provides the number of genes overlapping expressed sequence alignments for different transcript libraries. **Table S5,** provides counts of near-universal insect orthologous groups that are missing orthologs in each species. **Table S6.** provides evidence and sampling options used for the three AUGUSTUS gene sets AU9, AU11, and AU12. **Table S7.** provides gene prediction accuracy of GeneID and SGP2 on an *A. mellifera* artificial contig. **Figure S1.** shows the proportions of different transposable element groups in the *A. mellifera* genome. Click here for file

Additional file 2 Is a spreadsheet showing results of evaluation of 32 consensus gene sets generated with GLEAN. Click here for file

Additional file 3 Is a spreadsheet showing sources of biological evidence and other characteristics for each OGSv3.2 gene, and is the data used in comparing Type I New, Type II New and Previously Known genes. Click here for file

Additional file 4 Is a spreadsheet showing new venom proteins and known venom proteins with newly identified tryptic peptides in OGSv3.2. Click here for file

Additional file 5 Is a spreadsheet providing results of manually annotating venom peptides. Click here for file

Additional file 6 **Is a spreadsheet providing reciprocal best hit orthologs between *A. mellifera *** **and *D. melanogaster *** **and gene ontology for D. melanogaster proteins.** Click here for file

Additional file 7 Is a spreadsheet providing counts of InterPro domains in OGSv3.2 and OGSv1.0. Click here for file

Additional file 8 Is a spreadsheet providing an analysis of 1234 OGSv3.2 genes that overlap interspersed repeats identified by REPET. Click here for file
