# Supplementary material for: Finding the missing honey bee genes: lessons learned from a genome upgrade
Source: BMC Genomics. 2014 Jan 30;15:86. doi: 10.1186/1471-2164-15-86 (PMC4028053; doi:10.1186/1471-2164-15-86)

**Supplemental Tables**

**Table S1. Additional Sequence Data Details**

| SRA Run Number | Number of Spots | Number of Reads | Number of Bases | Library Type |
| --- | --- | --- | --- | --- |
| SRR000297 | 259,377 | 518,754 | 75,104,764 | Fragment |
| SRR000298 | 328,596 | 657,192 | 93,879,351 | Fragment |
| SRR000299 | 273,533 | 547,066 | 79,202,614 | Fragment |
| SRR019130 | 282,422 | 564,844 | 81,367,322 | Fragment |
| SRR019131 | 279,232 | 558,464 | 80,872,686 | Fragment |
| SRR019132 | 404,965 | 1,619,860 | 46,669,227 | Paired-End |
| SRR019133 | 509,572 | 2,038,288 | 58,797,274 | Paired-End |
| SRR019134 | 321,611 | 643,222 | 92,896,583 | Fragment |
| SRR019135 | 244,506 | 489,012 | 71,047,488 | Fragment |
| SRR019136 | 336,715 | 673,430 | 97,164,783 | Fragment |
| SRR019137 | 152,360 | 304,720 | 44,158,971 | Fragment |
| SRR019138 | 278,976 | 557,952 | 80,603,766 | Fragment |
| SRR019139 | 136,292 | 272,584 | 39,797,442 | Fragment |
| SRR019140 | 394,686 | 1,578,744 | 188,910,443 | Paired-End |
| SRR343151 | 44,973,259 | 89,946,518 | 4,497,325,900 | SOLiD |
| Total | 49,176,102 | 100,970,650 | 5,627,798,614 |  |

**Table S2. New and Previously Known OGSv3.2 genes with relaxed mapping criteria**. Genes were mapped to Amel_2.0 assembly with relaxed mapping criteria of 50% gene coverage and 95% identity. Biological evidence includes transcript overlap (spliced or un-spliced), peptide hit, protein homolog alignment overlap, or InterPro domain presence.

|  | | **All OGSv3.2** | **Type I New Genes** | **Type II New Genes** | **Previously Known Genes** |
| --- | --- | --- | --- | --- | --- |
| Number of genes (% of total OGSv3.2 genes) | | 15314 (100%) | 377 (2.5%) | 4081 (26.6%) | 10856 (70.9%) |
| Scaffold Analysis | Number of Genes within Mapped Scaffolds (% of no. of gene type) | 13285 (86.8%) | 252 (66.8%) | 3288 (80.6%) | 9745 (89.8%) |
|  | Number of Genes within Un-mapped Scaffolds (% of no. of gene type) | 2029 (13.2%) | 125 (33.2%) | 793 (19.4%) | 1111 (10.2%) |
| CDS Analysis | Average CDS Length | 1266.1 | 677.7 | 347.9 | 1631.6 |
|  | Average No. CDS Exons | 5.3 | 3.5 | 2.2 | 6.6 |
|  | Number of Single CDS Exon Genes (% of no. of gene type) | 2059 (13.4%) | 99 (26.3%) | 1240 (30.4%) | 720 (6.6%) |
|  | Number of Multi-CDS Exon Genes (% of no. of gene type) | 13255 (86.6%) | 278 (73.7%) | 2841 (69.6%) | 10136 (93.4%) |
| Intron Analysis | Number of Introns (% of total OGSv3.2 introns) | 66212 (100%) | 929 (1.4%) | 4795 (7.2%) | 60488 (91.4%) |
|  | Number of Introns Validated by EST Intron Coordinates (% of introns of gene type) | 54514 (82.3%) | 547 (58.9%) | 2201 (45.9%) | 51766 (85.6%) |
| Peptide Analysis | Number of genes with a peptide match (% of no. of gene type) | 3631 (23.7%) | 35 (9.3%) | 95 (2.3%) | 3501 (32.2%) |
| Protein Analysis | No. of genes with overlap to at least one protein alignment (% of no. of gene type) | 6778 (44.3%) | 71 (18.8%) | 210 (5.1%) | 6497 (59.8%) |
|  | No. of genes with overlap to a Dmel protein alignment (% of no. of gene type) | 1205 (7.9%) | 11 (2.9%) | 15 (0.4%) | 1179 (10.9%) |
| Total Spliced and Un-Spliced Expressed Sequence Support | No. of genes with overlap to at least one transcript alignment from any of the ten libraries (% of no. of gene type) | 13517 (88.3%) | 323 (85.7%) | 2883 (70.6%) | 10311 (95.0%) |
| Spliced Expressed Sequence Analysis | No. of genes with overlap to at least one transcript alignment from each of the ten libraries (% of no. of gene type) | 1062 (6.9%) | 6 (1.6%) | 17 (0.4%) | 1039 (9.6%) |
|  | No. of genes with overlap to at least one transcript alignment from any of the ten libraries (% of no. of gene type) | 12172 (79.5%) | 264 (70%) | 2205 (54%) | 9703 (89.4%) |
|  | No. of genes without overlap to any transcript alignments in any of the ten libraries (% of no. of gene type) | 3142 (20.5%) | 113 (30%) | 1876 (46%) | 1153 (10.6%) |
|  | Genes broadly expressed across four tissues (% of no. of gene type) | 2326 (15.2%) | 21 (5.6%) | 98 (2.4%) | 2207 (20.3%) |
|  | Genes narrowly expressed in only a single tissue (% of no. of gene type) | 3346 (21.8%) | 102 (27.1%) | 1190 (29.2%) | 2054 (18.9%) |
|  | No. of genes without overlap to any transcript alignments in any of the four tissues (% of no. of gene type) | 3632 (23.7%) | 132 (35%) | 2023 (49.6%) | 1477 (13.6%) |
| Analysis of Alignments to Other Bee Genomes | No. of genes that align to Aflo_1.0 (% of no. of gene type) | 13491 (88.1%) | 188 (49.9%) | 2686 (65.8%) | 10617 (97.8%) |
|  | No. of genes that align to Bter_1.0 (% of no. of gene type) | 12262 (80.1%) | 159 (42.2%) | 1660 (40.7%) | 10443 (96.2%) |
| Evidence Supported Genes | No. of genes with overlap to at least one form of biological evidence (% of no. of gene type) | 14084 (92.0%) | 325 (86.2%) | 3043 (74.6%) | 10716 (98.7%) |
|  | No. of genes that align to Aflo_1.0 and/or Bter_1.0 and/or overlap at least one form of biological evidence (% of no. of gene type) | 14836 (96.9%) | 338 (89.7%) | 3674 (90.0%) | 10824 (99.7%) |
| GC Analysis | Number of genes on GC compositional domains >10kb (% of OGSv3.2 total) | 15224 (99.4%) | 373 (2.5%) | 4051 (26.6%) | 10800 (70.9%) |
|  | Avg. GC Content of Compositional Domain Gene Resides in | 29.60% | 28.70% | 31.80% | 28.70% |
| ENC Analysis | Effective Number of Codons | 44.95 | 38.82 | 45.63 | 44.91 |

**Table S3. Canonical versus non-canonical intronic splice site sequence analysis for OGSv3.2.** Genes mapped to Amel_2.0 assembly with stringent mapping criteria of 80% gene coverage and 95% identity.

|  | All OGSv3.2 | Type I New Genes | Type II New Genes | Previously Known Genes |
| --- | --- | --- | --- | --- |
| Total introns (% of total OGSv3.2 introns) | 66212 (100%) | 3585 (5.4%) | 4333 (6.5%) | 58294 (88.0%) |
| Canonical introns (% of no. of gene type) | 65669 (99.2%) | 3537 (98.7%) | 4305 (99.4%) | 57827 (99.2%) |
| Non-canonical introns (% of no. of gene type) | 543 (0.8%) | 48 (1.3%) | 28 (0.6%) | 467 (0.8%) |
| Introns supported by transcript alignment (% of no. of gene type) | 54514 (82.3%) | 2573 (71.8%) | 1930 (44.5%) | 50011 (85.8%) |
| Introns not supported by transcript alignment (% of no. of gene type) | 11698 (17.7%) | 1012 (28.2%) | 2403 (55.5%) | 8283 (14.2%) |
| Canonical, supported introns (% of no. of supported introns for gene type) | 54145 (99.3%) | 2551 (99.1%) | 1916 (99.3%) | 49678 (99.3%) |
| Non-canonical, supported introns (% of no. of supported introns for gene type) | 369 (0.7%) | 22 (0.9%) | 14 (0.7%) | 333 (0.7%) |

**Table S4. OGSv3.2 Genes Overlapping Expressed Sequence Alignments**

|  | Number of genes overlapped by a transcript in the given set | % total OGSv3.2 genes (15,314) |
| --- | --- | --- |
| Spliced_abdomen_contig | 4,408 | 28.8% |
| Unspliced_abdomen_contig | 1,799 | 11.7% |
| Abdomen | 5,413 | 35.3% |
| Spliced_brain_ovary_contig | 7,340 | 47.9% |
| Unspliced_brain_ovary_contig | 2,105 | 13.7% |
| Brain_ovary | 8,437 | 55.1% |
| Spliced_embryo_contig | 5,956 | 38.9% |
| Unspliced_embryo_contig | 1,388 | 9.1% |
| Embryo | 6,673 | 43.6% |
| Spliced_forager_brain contig | 10,198 | 66.6% |
| Unspliced_forager_brain contig | 6,725 | 43.9% |
| Forager brain | 12,134 | 79.2% |
| Spliced_larvae_contig | 3,960 | 25.9% |
| Unspliced_larvae_contig | 707 | 4.6% |
| Larvae | 4,335 | 28.3% |
| Spliced_mixed_antennae_contig | 4,088 | 26.7% |
| Unspliced_mixed_antennae_contig | 971 | 6.3% |
| Mixed_antennae | 4,578 | 29.9% |
| Spliced_NCBI_EST_contig | 5,983 | 39.1% |
| Unspliced_NCBI_EST_contig | 3,935 | 25.7% |
| NCBI_EST | 7,320 | 47.8% |
| Spliced_nurse_brain contig | 10,111 | 66.0% |
| Unspliced_nurse_brain contig | 6,549 | 42.8% |
| Nurse brain | 11,959 | 78.1% |
| Spliced_ovary_contig | 7,926 | 51.8% |
| Unspliced_ovary_contig | 1,570 | 10.3% |
| Ovary | 8,698 | 56.8% |
| Spliced_testes_contig | 3,927 | 25.6% |
| Unspliced_testes_contig | 833 | 5.4% |
| Testes | 4,332 | 28.3% |

**Table S5. Counts of near-universal insect orthologous groups that are missing orthologs in each species.** Total counts were partitioned into groups with only single-copy orthologs (SC) and those with gene duplications (PR), further divided into those with only one missing species (“allbut1” )and those with two missing species (“allbut2”).

| **Species** | **SC-allbut1** | **SC-allbut2** | **PR-allbut1** | **PR-allbut2** | **Totals** |
| --- | --- | --- | --- | --- | --- |
| ***Pediculus humanus*** | 104 | 151 | 102 | 116 | 473 |
| ***Acyrthosiphon pisum*** | 230 | 218 | 114 | 118 | 680 |
| ***Nasonia vitripennis*** | 91 | 76 | 70 | 39 | 276 |
| ***Apis mellifera V3.2*** | 27 | 37 | 23 | 25 | 112 |
| ***Apis mellifera pre_release2*** | 80 | 74 | 65 | 44 | 263 |
| ***Linepithema humile*** | 17 | 48 | 18 | 41 | 124 |
| ***Pogonomyrmex barbatus*** | 49 | 37 | 21 | 41 | 148 |
| ***Tribolium castaneum*** | 91 | 93 | 61 | 40 | 285 |
| ***Danaus plexippus*** | 115 | 112 | 55 | 45 | 327 |
| ***Anopheles gambiae*** | 99 | 172 | 84 | 89 | 444 |
| ***Drosophila melanogaster*** | 98 | 172 | 60 | 90 | 420 |

**Table S6: Evidence and sampling options used for the three AUGUSTUS gene sets AU9, AU11, and AU12.**

|  | AU9 | AU11 | AU12 |
| --- | --- | --- | --- |
| Hints from RNA-seq data | X | X | X |
| Hints from ESTs | X | X | X |
| Hints from Peptides |  |  | X |
| Alternative transcripts predicted from extrinsic evidence | X | X | X |
| Alternative transcripts predicted from sampling |  | X |  |

**Table S7. Accuracy of gene prediction on an *A. mellifera* artificial contig** consisting of 431 concatenated melon test sequences with approximately 800 nucleotides of sequence between each of the gene models using the *ab initio* program GeneID. The accuracy of SGP2 (homology evidence-based prediction tool that used the *N. giraulti*, *N. longicornis* and *N. vitripennis* genome as reference) was also tested for accuracy on the same set of sequences (SN & SP: sensitivity & specificity at nucleotide level; SNe & SPe: sensitivity & specificity at exon level; SNg & SPg: sensitivity & specificity at gene level).

| Program/Parameter | SN | SP | SNe | SPe | SNg | SNp |
| --- | --- | --- | --- | --- | --- | --- |
| GeneID Bee | 0.95 | 0.96 | 0.80 | 0.82 | 0.38 | 0.33 |
| SGP2 Bee (*Nasonia* spp.) | 0.96 | 0.97 | 0.82 | 0.83 | 0.41 | 0.42 |

**Supplemental Figure**

**Figure S1. Elements by proportion (compared to all elements)**

*Apis mellifera*, blue: LTR-retro-transposons, orange: non-LTR-retro-transposons, blue: DNA transposons, green: non-interspersed repeats, grey: elements that are unclassified (at different levels).


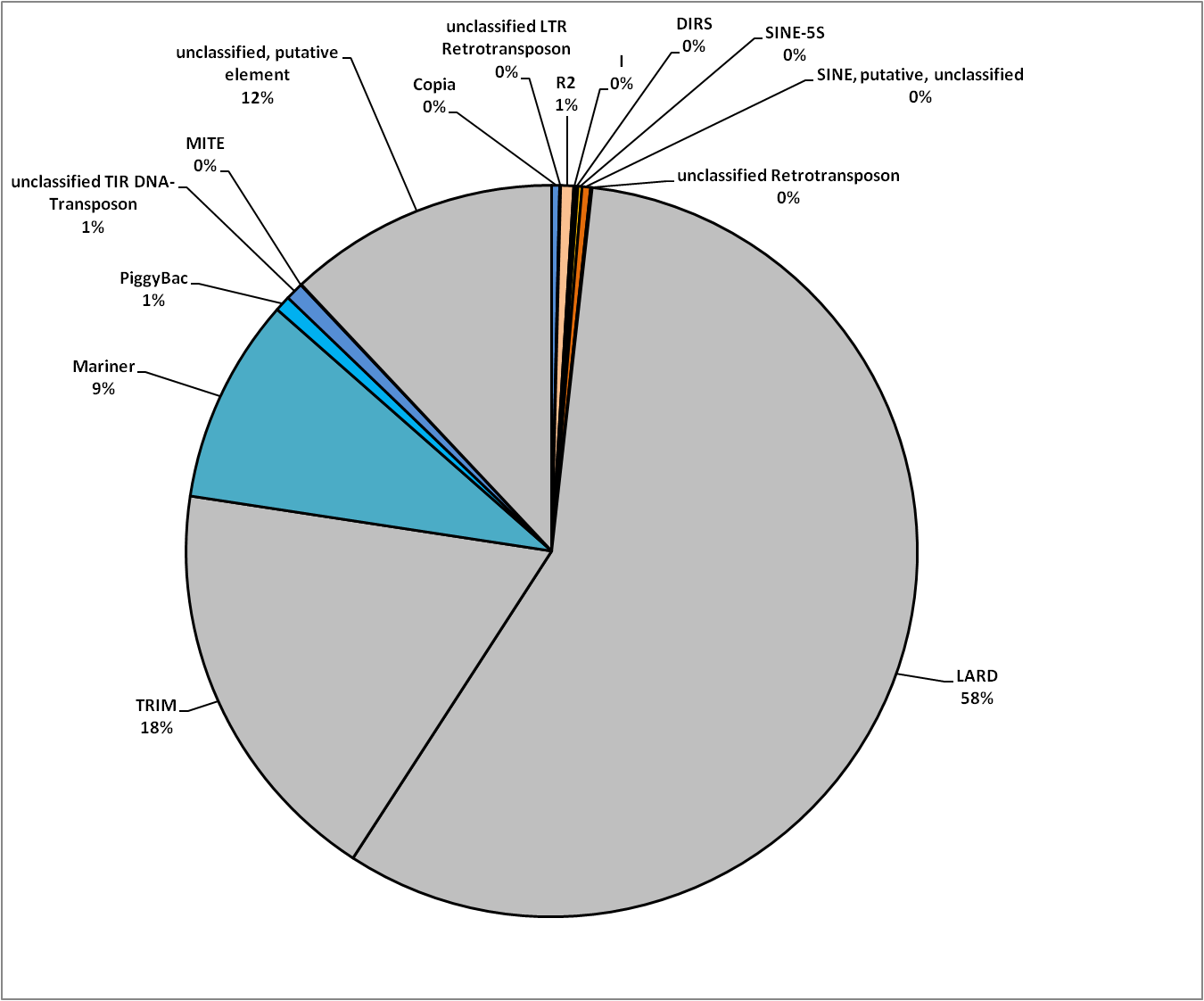

Supplement: Additional file 1 — Is a document containing Tables S1 through S7 and Figure S1. Table S1. provides details for genomic sequencing runs, listed by SRA run number. Table S2. provides a comparison of New and Previously Known OGSv3.2 genes based on relaxed mapping criteria. Table S3. provides a comparison of frequencies of canonical and non-canonical splice sites in New and Previously Known genes. Table S4. provides the number of genes overlapping expressed sequence alignments for different transcript libraries. Table S5, provides counts of near-universal insect orthologous groups that are missing orthologs in each species. Table S6. provides evidence and sampling options used for the three AUGUSTUS gene sets AU9, AU11, and AU12. Table S7. provides gene prediction accuracy of GeneID and SGP2 on an A. mellifera artificial contig. Figure S1. shows the proportions of different transposable element groups in the A. mellifera genome. [file 1471-2164-15-86-S1.docx]
